# The Relationship between Membrane Potential and Calcium Dynamics in Glucose-Stimulated Beta Cell Syncytium in Acute Mouse Pancreas Tissue Slices

**DOI:** 10.1371/journal.pone.0082374

**Published:** 2013-12-06

**Authors:** Jurij Dolenšek, Andraž Stožer, Maša Skelin Klemen, Evan W. Miller, Marjan Slak Rupnik

**Affiliations:** 1 Institute of Physiology, Faculty of Medicine, University of Maribor, Maribor, Slovenia; 2 Centre for Open Innovations and Research, University of Maribor, Maribor, Slovenia; 3 Department of Pharmacology, University of California at San Diego, La Jolla, California, United States of America; 4 Centre of Excellence for Integrated Approaches in Chemistry and Biology of Proteins, Ljubljana, Slovenia; University of Szeged, Hungary

## Abstract

Oscillatory electrical activity is regarded as a hallmark of the pancreatic beta cell glucose-dependent excitability pattern. Electrophysiologically recorded membrane potential oscillations in beta cells are associated with in-phase oscillatory cytosolic calcium activity ([Ca^2+^]_i_) measured with fluorescent probes. Recent high spatial and temporal resolution confocal imaging revealed that glucose stimulation of beta cells in intact islets within acute tissue slices produces a [Ca^2+^]_i_ change with initial transient phase followed by a plateau phase with highly synchronized [Ca^2+^]_i_ oscillations. Here, we aimed to correlate the plateau [Ca^2+^]_i_ oscillations with the oscillations of membrane potential using patch-clamp and for the first time high resolution voltage-sensitive dye based confocal imaging. Our results demonstrated that the glucose-evoked membrane potential oscillations spread over the islet in a wave-like manner, their durations and wave velocities being comparable to the ones for [Ca^2+^]_i_ oscillations and waves. High temporal resolution simultaneous records of membrane potential and [Ca^2+^]_i_ confirmed tight but nevertheless limited coupling of the two processes, with membrane depolarization preceding the [Ca^2+^]_i_ increase. The potassium channel blocker tetraethylammonium increased the velocity at which oscillations advanced over the islet by several-fold while, at the same time, emphasized differences in kinetics of the membrane potential and the [Ca^2+^]_i_. The combination of both imaging techniques provides a powerful tool that will help us attain deeper knowledge of the beta cell network.

## Introduction

Beta cells within an islet of Langerhans are coupled into an insulin-secreting syncytium and this coupling critically affects their function [1]. The nature and extent of coupling have been assessed in isolated islets with a number of different experimental approaches. Morphological studies using electron microscopy revealed that beta cells are interconnected via gap junctions [2]. Molecular biological tools showed that gap junctions are made of the connexin 36 protein in rats [3], mice [4,5], and in humans [6]. Dye-injection studies using sharp electrodes indicated restricted communication between different beta cells, with the dye injected into one cell remaining restricted to the impaled cell [7] or spreading to only a few other neighboring cells [8]. Since the dye transfer depends heavily on its charge and size, interpretation of these data is difficult [9]. Electrophysiological studies have been used to observe normal electrical activity of beta cells [10,11] or the effect on normal electrical activity of current injected into one of the impaled cells [12]. Similarly to dye-injection studies, current-injection studies suggested only limited coupling between beta cells, with potential changes being detectable over a distance of less than 35 micrometers (diameter of a few cells) [12]. The complex three dimensional architecture of the islet tissue and the low membrane input resistance due to open K_ATP_ channels make membrane depolarization by an injected current highly inefficient [13]. Coupling between physically closest beta cells was also studied with the patch-clamp technique on isolated cell pairs [14], in whole isolated islets [7] as well as in tissue slices [15]. These latter studies clearly confirmed electrical coupling between neighboring beta cells and enabled estimation of the size of gap junctional conductance and the number of directly coupled cells, but they did not allow any direct conclusions regarding coupling of physically more separated cells [7,14,15]. Calcium imaging experiments on isolated islets using CCD cameras [16-19] and confocal microscopy [20,21] further contributed to our understanding of coupling between beta cells. High spatial and temporal resolution confocal microscopy has been recently used to assess coupling also in islets in acute pancreas tissue slices [22,23].

Several lines of evidence support the view that electrical activity spreads over islets of Langerhans in the form of directed waves of membrane potential oscillations. A close comparison of electrical activity and changes in intracellular concentration of calcium ions ([Ca^2+^]_i_) in beta cells stimulated by glucose showed that the bursts of electrical activity have the same frequency, are in phase and of similar shape not only in pairs of cells lying close together [10] but even in pairs of cells separated by greater distances, such as the whole diameter of an islet, suggesting an efficient electrical coupling [16,17,20,21,24]. More specifically, in two simultaneously monitored cells the phase difference of bursts, assessed by the time delay between the first spikes of bursts, was shown to increase with the Euclidean distance between the two cells. Furthermore, in the majority of pairs, the same cell was shown to lead the pace over long time periods [24]. A study employing extracellular recordings at two points on isolated islets pointed out the possibility that membrane potential oscillation propagates across islets in the form of waves with a velocity of approximately 100 μm/s [25]. Since in beta cells the electrical activity is believed to be closely synchronized with changes in [Ca^2+^]_i_ [19], the abovementioned view is further supported by the results of calcium imaging experiments showing that [Ca]_i_ oscillations are well synchronized among different parts [17,19] and individual beta cells of an islet [20,21], and that [Ca]_i_ oscillations spread in a regular manner across islets of Langerhans with approximately the same velocity as excitation [18,22]. 

In contrast to calcium imaging, however, to date changes in membrane potential have never been directly assessed on a large number of beta cells simultaneously with sufficient temporal resolution to allow detection of possible short temporal differences in membrane potential changes between individual cells. A study employing fluorescence resonance energy transfer-based voltage probe in isolated islets showed that all beta cells of an islet of Langerhans display similar time-dependent changes in membrane potential, but the temporal resolution used in this study (3 Hz) was too low to enable detection of possible depolarization waves [26]. Additionally, this study also reported a tight coupling of membrane potential changes in beta cells from all layers of an islet with [Ca^2+^]_i_ changes in beta cells from the periphery of the same islet [26]. Again, the temporal resolution employed did not enable comparison of membrane potential and [Ca^2+^]_i_ changes with sufficient temporal detail to quantify possible differences between the two signals at the level of an individual cell and the islet as a whole. 

Therefore, the evidence for waves of membrane potential oscillations and the extent of coupling between [Ca^2+^]_i_ and membrane potential oscillations at the level of many beta cells is only circumstantial and it remains unclear (i) to what extent the electrical and calcium signals are temporally synchronized, (ii) whether detectable waves of membrane potential oscillations spread across islets, and (iii) whether they do so in the same direction and with a comparable velocity as waves of [Ca^2+^]_i_ changes. 

To address these questions that are fundamental to our understanding of the beta cell syncytium as well as other comparable syncytia, we combined the tissue slice method [27] with live cell imaging employing a new generation of voltage-sensitive dyes [28]. The tissue slice method was shown to be compatible with tissue handling required for partitioning of fluorescent dyes into plasma membrane (to measure membrane potential) and for enabling the access of AM-based fluorescent dyes into cell interior (to measure [Ca^2+^]_i_) of a large number of cells in an islet´s cross-section [22,23]. This is a prerequisite to detect and analyze waves spreading through an islet. The new generation of voltage-sensitive dyes showed voltage-sensitivity and temporal characteristics that should enable detection and resolution of bursts of electrical activity, characteristic of beta cells. To address the relationship between membrane potential and [Ca^2+^]_i_ dynamics into more detail, we also performed whole-cell patch-clamp experiments, [Ca^2+^]_i_ imaging experiments and finally double staining of tissue slices with both voltage- and calcium-sensitive dyes. 

## Materials and Methods

### Ethics statement

The study was conducted in strict accordance with all national and European recommendations pertaining to work with experimental animals, and all efforts were made to minimize suffering of animals. The protocol was approved by the Administration of the Republic of Slovenia for Food Safety, Veterinary and Plant Protection (permit number: 34401-61-2009/2). 

### Tissue slice preparation

Tissue slices were cut from pancreata of 10-20 week old NMRI mice of either sex as described in detail previously [27]. In brief, after sacrificing the animals by cervical dislocation, the abdomen was exposed via laparotomy and low-melting point 1.9% agarose (Lonza Rockland Inc., Rockland, Maine, USA) in extracellular solution (ECS, consisting of (in mM) 125 NaCl, 26 NaHCO_3_, 6 glucose, 6 lactic acid, 3 myo-inositol, 2.5 KCl, 2 Na-pyruvate, 2 CaCl_2_, 1.25 NaH_2_PO_4_, 1 MgCl_2_, 0.5 ascorbic acid) at 40°C was injected into the proximal common bile duct clamped at the papilla of Vater. Immediately thereafter, the pancreas was cooled by pouring over it an ice-cold ECS. Then the agarose-injected pancreas was extracted and gently washed in ice-cold ECS, small blocks of tissue (0.1-0.2 cm^3^ in size) were cut and finally transferred to a 5 ml Petri dish filled with agarose at 40°C. Individual cubes of cooled agarose containing tissue blocks were cut from hardened agarose and glued (Super Attak, Henkel Slovenija d.o.o., Maribor, Slovenia) onto the sample plate of the VT 1000 S vibratome (Leica, Nussloch, Germany). The tissue was cut at 0.05 mm s^-1^ and 70 Hz into 140 µm-thick slices of a surface area of 20-100 mm^2^. Throughout preparation and during slicing the tissue was held in an ice-cold ECS continuously bubbled with a gas mixture containing 95% O_2_ and 5% CO_2_ at barometric pressure to ensure oxygenation and a pH of 7.4. After cutting the slices were collected in 30 ml of HEPES-buffered saline at room temperature (HBS, consisting of (in mM) 150 NaCl, 10 HEPES, 6 glucose, 5 KCl, 2 CaCl_2_, 1 MgCl_2_; titrated to pH=7.4 using 1 M NaOH) and then incubated in the dye-loading solution. All chemicals were obtained from Sigma-Aldrich (St. Louis, Missouri, USA) unless indicated. 

### Loading of dyes

For [Ca^2+^]_i_ imaging 10-12 slices were incubated in a Petri dish (5 ml) filled with 3.333 ml of HBS containing 6 μM Oregon Green 488 BAPTA-1 AM calcium dye (OGB-1, Invitrogen, Eugene, Oregon, USA), 0.03% Pluronic F-127 (w/v) and 0.12% dimethylsulphoxide (DMSO, v/v) for 50 minutes on an orbital shaker (50 turns min^-1^) at room temperature and protected from light. Imaging was made within 12 hours after staining. Following staining, slices were kept protected from light in a dye-free HBS which was exchanged every two hours. Fluorescence intensity changes were analyzed and presented as described previously [22]. 

For membrane potential measurements up to 4 slices were incubated in a Petri dish (5 ml) filled with 3 ml of HBS containing 1.3 μM VoltageFluor (VF2.1) dye [28], 0.12% Pluronic F-127 (w/v) and 0.55% DMSO (v/v) for 50 minutes on an orbital shaker (50 turns min^-1^) at room temperature and protected from light. Due to dye internalization, imaging was done within an hour after staining. 

For simultaneous measurements of membrane potential and [Ca^2+^]_i_ oscillations, up to 4 slices were incubated in a Petri dish (5 ml) filled with 3 ml of HBS containing 1.3 μM VoltageFluor (VF2.1) dye and 7.4 μM Rhod-2 AM calcium dye (Rhod-2, Invitrogen, Eugene, Oregon, USA), 0.12% Pluronic F-127 (w/v) and 0.55% DMSO (v/v) for 45 minutes on an orbital shaker (50 turns min^-1^) at room temperature and protected from light. Rhod-2 was preferred in the double staining experiments due to its spectral properties that enabled discrimination of its signal from the VF fluorescent signal (Figure S4). To substantiate the usage of Rhod-2, which can get internalized into mitochondria [29], as a calcium indicator of the cytosolic [Ca^2+^] and to make sure that it performs similarly to OGB-1 used in other experiments, we performed control experiments in which slices were stained with both Rhod-2 and OGB-1. Traces reported by the two indicators were found to be practically identical (Figure S5 and Text S1). Imaging was completed within an hour after staining. 

### Imaging of [Ca^2+^]_i_ and membrane potential oscillations

For the measurement of [Ca^2+^]_i_ or membrane potential changes in conjunction with the patch-clamp electrophysiology, the imaging was done with a water-cooled CCD camera Andor DV 887AC-FI (Ixon, Andor Technology, Belfast, UK) mounted on an upright Nikon Eclipse E600 FN microscope (Nikon, Tokyo, Japan). OGB-1 was excited at 488 nm and VF at 500 nm with a monochromator (Polychrome IV, TILL Photonics). Monochromatic light was reflected by a dichroic mirror (405 nm) and directed through a 60x water immersion objective (NA = 1.0). The emitted fluorescence was transmitted by the dichroic mirror and further filtered through a 520-nm long pass filter. Images (256 x 256 pixels) were taken at a frequency of 2 Hz (light exposure time 100 ms per image) for the [Ca^2+^]_i_ and at a frequency of 5 Hz (light exposure time 100 ms per image) for the VF. 

Confocal [Ca^2+^]_i_ imaging using OGB-1 was performed on a Leica TCS SP5 AOBS Tandem II upright confocal system using a Leica HCX APO L 20x water immersion objective (NA = 1.0). OGB-1 was excited by an argon 488 nm laser and the emitted fluorescence was detected by Leica HyD detector in the range of 500–700 nm (all from Leica Microsystems GmbH). Individual slices were imaged in a temperature-controlled bath chamber mounted on the microscope (37°C, Luigs & Neumann) continuously perifused with ECS. Sampling rate was 20-50 Hz at 256 x 64 pixels.

Both the membrane potential imaging and simultaneous imaging of the membrane potential and [Ca^2+^]_i_ were performed on a Leica TCS SP5 II inverted confocal system using a Leica HCX PL APO CS 20x immersion objective (NA = 0.7). VF and Rhod-2 were excited by an argon 514 nm and a diode pumped solid state (DPSS) 561 nm lasers, respectively. Fluorescence was detected by Leica HyD detectors (all from Leica Microsystems GmbH) in the range of 520-554 nm for VF and 570-700 nm for Rhod-2. In the bath chamber, the slice was continuously perifused with the bubbled ECS at 35 -37 °C. Sampling rate was 5-172 Hz at either 512 x 256 pixels or 512 x 128 pixels (binned 2x2 or 4x4 for higher frequency sampling). To confirm that VF followed the voltage changes in beta cells, we performed a control experiment measuring membrane potential from the whole-cell patch-clamped cell while simultaneously measuring VF signal from the neighboring cells (Figure S6). Since the spreading of the membrane depolarization is fast enough to mask any differences between neighboring cells at the temporal resolution achievable in these experiments, the neighboring cells reflected fluorescence signal of the patch-clamped cell very well. These experiments confirmed that the change in the VF fluorescence corresponded to electrical changes as expected from what we showed in previous systems (HEK cells, rat neurons, intact leech ganglia) [30].

The slices were fixed to the bottom of the bath chamber by an U-shaped platinum weight with nylon-fibers. To avoid recording from cells in the damaged cut surface, cells lying at least 15 μm below the surface were imaged. The optical section thickness was calculated at 4 μm for the upright and 4-7 μm for the inverted confocal setup. The chosen thickness of the optical section, the acquisition frequency and the binning gave a reasonable trade-off between a satisfactory signal strength at the lowest acceptable laser power (to avoid photo-bleaching and prolong the maximum time of recording) on one hand and the need to keep the optical sections as thin as possible. Before and after recording a time series, a high spatial resolution fluorescence image was taken and used as a reference to assess motion artifacts and regions of interest (ROIs) for the off-line analyses of dynamics.

### Whole-cell patch-clamp measurements of membrane potential oscillations

Patch pipettes were pulled from borosilicate glass capillaries (GC150F-15, Harvard Apparatus, USA) using a horizontal pipette puller (P-97, Sutter Instruments, USA). The pipette resistance was 2–3 MΩ in K^+^-based solution. Fast pipette capacitance (C_fast_), slow membrane capacitance (C_slow_) and series conductance (G_s_) were compensated accordingly. Only experiments with G_s_>50 nS were analyzed. Recordings were performed in the standard whole-cell mode via a patch-clamp lock-in amplifier (SWAM IIc, Celica, Slovenia) connected to a PC via A/D converter (16 bit, NI USB-6341, X Series Multifunction DAQ, National Instruments, USA) and recorded on the PC hard disk using WinFluor V3.4.1 software (John Dempster, University of Strathclyde, UK) at a sampling rate of 1 kHz. The same software was used to apply voltage protocol for identifying beta cells by their Na^+^ current inactivation properties [31] and for measuring membrane potential oscillations during stimulation with glucose. The pipette solution used for membrane potential measurements was composed of (in mM) 125 potassium methanesulfonate, 20 KCl, 40 HEPES, 2 MgCl_2_, 5 Na_2_ATP, titrated to pH = 7.2 using 1 M KOH. Osmolality of the intracellular and all extracellular solutions was 300±10 mOsm.

### Data Analysis

Membrane potential and [Ca^2+^]_i_ oscillations were analyzed off-line. ROIs were selected as indicated in Figure S4 (for membrane potential detection ROIs included cell membrane as well as its interior and for [Ca^2+^]_i_ detection cell interior only, as indicated in Figures S4B and S4C, respectively). Cells within the islet were discriminated functionally: beta cells do not display any oscillatory activity in the low glucose regime, but activate in response to stimulation with high glucose [32,33]. Figure S1 shows a typical temporal shape of the beta cell membrane potential response to high glucose and the part of the response where the high temporal resolution analyses that are the cornerstone of our present study were performed is indicated. Time series were exported employing Leica Application Suite Advanced Fluorescence software (LASAF, Leica Microsystems GmbH). Further analysis was performed using custom-made scripts in the MATLAB program (The MathWorks, Inc.) and Image J. Photo-bleaching was accounted for by a combination of linear and exponential fit as described previously [22]. The fluorescence signals were expressed as F_t_/F_0t_ - 1, F_t_ being the fluorescence signal recorded at an individual time point during the experiment and F_0t_ being the basal fluorescence signal at an individual time point estimated from the fit, respectively. 

Membrane potential traces were smoothed with a moving average filter with local regressions calculated using weighted linear least squares and a 2^nd^ degree polynomial model. Smoothing was minimized in order to not alter the shape of oscillations. The onset (start_50_) and end (end_50_) of an oscillation (for both the membrane potential and [Ca^2+^]_i_ records in confocal setups) were determined from the duration at half maximal amplitude of an oscillation. To determine the wave-front velocities, we used start_50_ and end_50_ time references and respective Euclidean coordinates to plot vectors representing wave-front trajectories. Wave-front propagation was best represented by down-sampling the vector data to 1-20 Hz. Trajectory coordinates were calculated from the average coordinate of the group of cells in which the wave originated, and the average of the coordinates of consecutively activated groups of cells, respectively. [Ca^2+^]_i_ oscillation onset, recorded with the CCD camera, was determined as a deflection of the signal from the base line. Figures were plotted using the Matlab, SigmaPlot v11.0 (Systat Software, Inc) and CorelDRAW X5 (Corel Inc). 

Heterogeneous loading of cells with the calcium and membrane potential indicators could theoretically introduce artificial heterogeneity in the calcium and membrane potential responses between different cells. We believe that this was not of crucial importance in our study for two reasons. First, it is evident from the presented traces that qualitatively, the temporal profiles of membrane potential and [Ca^2+^]_I_ oscillations are of similar shape and second, there was no systematic correlation between the intensity of calcium indicator loading and the order of activation of different cells.

## Results

### Simultaneous whole-cell patch-clamp measurement of membrane potential oscillations and CCD camera based measurement of intracellular calcium concentration oscillations

**Figure 1:**
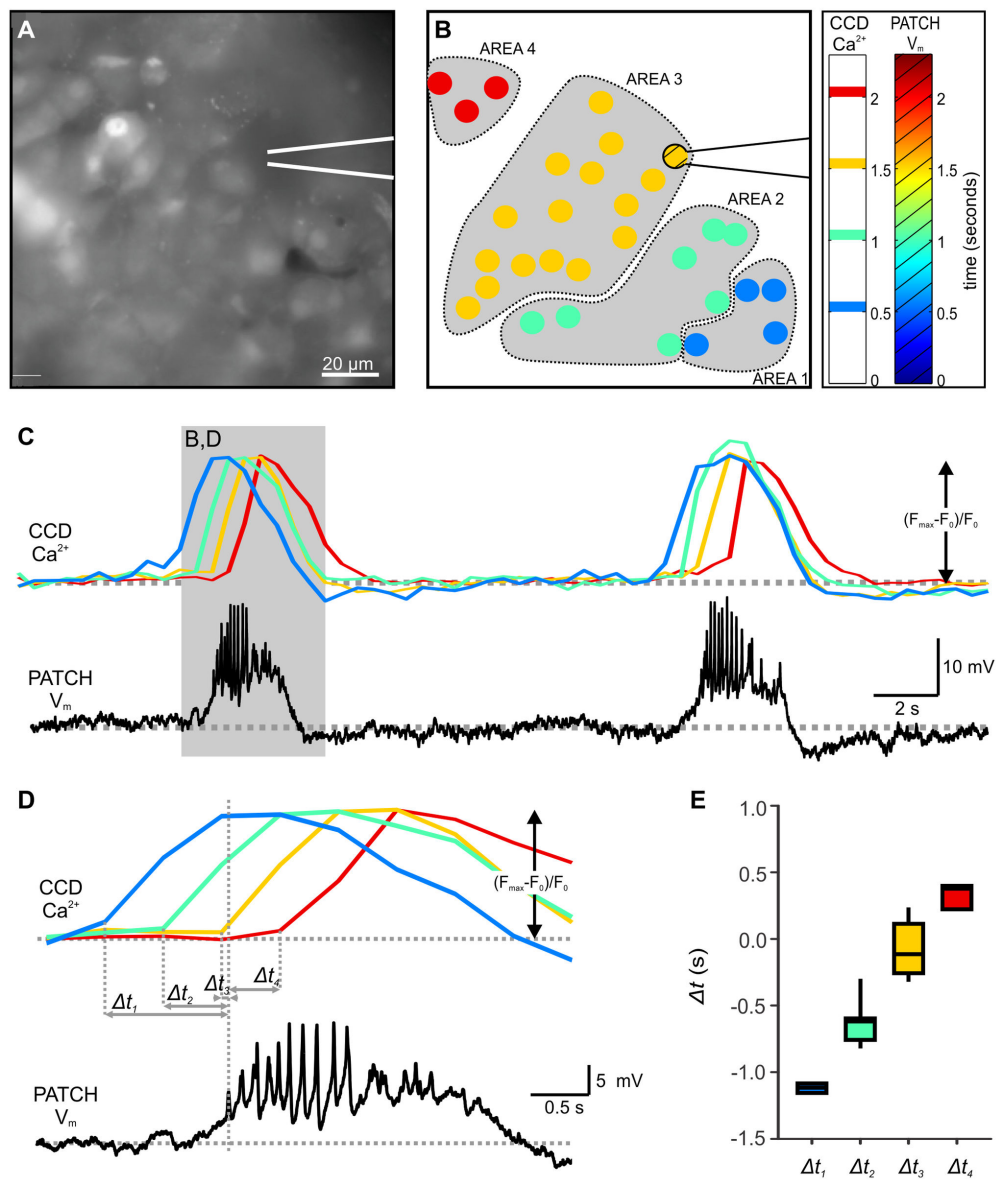
Simultaneous recording of membrane potential oscillations occurring in the patch-clamped cell and [Ca^2+^]_i_ oscillations occurring in other cells of the same islet of Langerhans. **A** [Ca^2+^]_i_ oscillations were measured with a CCD camera at a temporal resolution of 2 Hz in cells in the tissue slice loaded with OGB-1. The patch-clamp pipette position is indicated with white lines. **B** During stimulation with 12 mM glucose, [Ca^2+^]_i_ oscillations spread across the plane of the islet (from the lower right to the upper left corner in this figure). Responses of cells shown in A during a single [Ca^2+^]_i_ oscillation are color-coded to represent time lags between the onset of [Ca^2+^]_i_ increase in the cell where the visible part of the oscillation started and every other cell. Due to temporal resolution (2 Hz), cells were grouped into 4 groups only. The position of the patch-clamp pipette and the patch-clamped cell is indicated. The hatched pattern in the figure and legend bar indicates patch-clamp Data. **C** Two consecutive [Ca^2+^]_i_ (upper panel) and membrane potential (lower panel) oscillations during stimulation with glucose. Each [Ca^2+^]_i_ oscillation was accompanied by a membrane potential change consisting of a depolarization with superimposed spikes followed by a repolarization. Membrane potential was recorded at a temporal resolution of 1000 Hz. **D** A more detailed representation of the area indicated in C. The onset of high frequency spikes in the patched cell is temporally best correlated with the onset of [Ca^2+^]_i_ oscillation in the group of cells indicated in orange. For this group of cells the distance to oscillation origin was roughly the same as for the patched cell. E Time delays between the first spike in the patch-clamped cell and the onset of [Ca^2+^]_i_ oscillation in every other cell. Shortest time delays correspond to the orange group of cells for which the distance to wave origin was roughly the same as for the patch-clamped cell. Color coding as in B-D.

We first assessed the coupling between membrane potential and intracellular calcium concentration ([Ca^2+^]_i_) oscillations in the pancreatic tissue slices, previously described in isolated islets [17]. To this end, we simultaneously measured the [Ca^2+^]_i_ oscillations in responsive beta cells from a cross-sectional plane of an islet with a CCD camera and the membrane potential oscillations of a single beta cell of the same islet with the whole-cell patch-clamp technique. A single glucose stimulation protocol was used in this and other experiments. Extracellular solution containing 6 mM glucose was used as non-stimulatory condition, whilst raising the glucose concentration to 12 mM served to stimulate beta cells. Upon stimulation, beta cells responded in a characteristic way that allowed for their functional discrimination, with a transient period of continuous bursting accompanied by a transient increase in [Ca^2+^]_i_, followed by intermittent bursting accompanied by in-phase [Ca^2+^]_i_ oscillations superimposed on a sustained plateau of [Ca^2+^]_i_, as described previously [19,20,22]. In this study, we focused on oscillations and waves during the latter, stable phase of activity. In the islet in Figure 1, during stimulation with 12 mM glucose [Ca^2+^]_i_ oscillated at regular intervals (7 oscillations/min) (Figure 1C), with onsets of [Ca^2+^]_i_ oscillations spreading in a wave-like manner across the islet of Langerhans (Figure 1B). In accordance with this observation, time lags between the [Ca^2+^]_i_ oscillation in the cell where the oscillation appeared first and any other given cell depended on the spatial position of the cell (Figures 1B and 1C). In Figures 1B and 1C, four groups of cells are distinguished with respect to the onset of the [Ca^2+^]_i_ oscillation. These groups are rather discrete due to the low sampling frequency used in this setup (2 Hz for the CCD camera). The membrane potential oscillation recorded from a cell positioned approximately in the middle of the cross sectional area displayed typical bursts (slow oscillations with superimposed spikes) of electrical activity with a frequency identical to the frequency of [Ca^2+^]_i_ oscillations recorded in the surrounding cells. By comparing the timing of the first spike in the patch-clamped cell with onsets of [Ca^2+^]_i_ oscillations (Figure 1D), we found that as far as the temporal resolution permitted, the onset of electrical activity was tightly coupled to the [Ca^2+^]_i_ rise in a subset of beta cells whilst [Ca^2+^]_i_ oscillations in other cells either preceded or followed the rise in membrane potential, consistent with the presence of a calcium wave (note the equal pseudo-color in the patched cell and the group of cells whose [Ca^2+^]_i_ dynamics were best synchronized with the patched cell´s electrical activity in Figure 1B). [Ca^2+^]_i_ changes in the patch-clamped cell could not be assessed directly due to washout of the loaded dye into the pipette. We attempted to overcome this issue by inclusion of the dye into the patch-pipette, but this manipulation abolished the electrical activity measured by the whole-cell patch-clamp approach. However, from the [Ca^2+^]_i_ changes in cells lying at approximately the same distance from the origin of the wave, it seems plausible to suggest that in the patch-clamped cell, the membrane potential and [Ca^2+^]_i_ oscillations are synchronized better than are the [Ca^2+^]_i_ oscillations among different cells. If the same holds true for all cells in the islet, it is reasonable to hypothesize that a wave of membrane potential oscillation accompanies the wave of [Ca^2+^]_i_ oscillation. 

To confirm the latter assumption, to compare the duration and velocities of [Ca^2+^]_i_ and membrane potential oscillations and waves, and to finally determine which of the two waves temporally precedes the other, we resorted to confocal laser scanning imaging using calcium- and voltage-sensitive dyes. 

### Confocal [Ca^2+^]_i_ imaging

**Figure 2:**
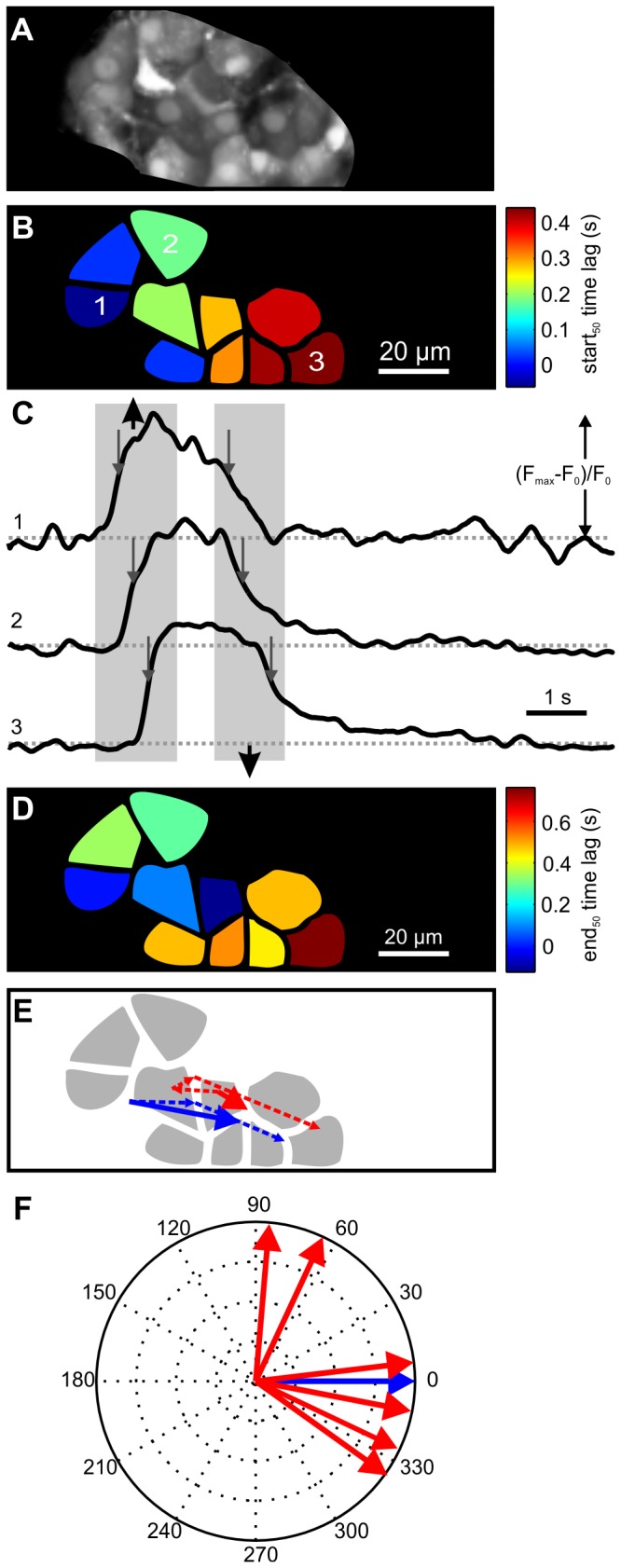
[Ca^2+^]_i_ oscillations during stimulation with 12 mM glucose. **A** A high resolution image of an islet of Langerhans showing cells loaded with the Oregon Green 488 BAPTA-1 (OGB-1) Ca^2+^ indicator. **B** Time lags between the start_50_ of [Ca^2+^]_i_ oscillation in the first and every other cell during stimulation with 12 mM glucose are color-coded. Note a wave-like spread of [Ca^2+^]_i_ increase from left to right in this focal plane. **C** A single oscillation from three cells indicated in B during stimulation with 12 mM glucose. Gray rectangular areas indicate time intervals for increase and decrease of [Ca^2+^]_i_ across different cells. Arrows indicate the duration of oscillations at their half-maximal amplitude. Y axis represents the normalized fraction of the difference between maximum and plateau baseline fluorescence. **D** A pseudo-colored representation of time lags between oscillation end_50_ in the cell in which [Ca^2+^]_i_ decreased first and every other cell. Note a wave-like spread of [Ca^2+^]_i_ decrease from left to right in this focal plane. **E** Trajectories describing the wave path of the oscillation start_50_ (blue, dashed line) and end_50_ (red, dashed line). The trajectories describing average directions of spreading are drawn with solid lines. Note that the wave of [Ca^2+^]_i_ decrease roughly follows the wave of [Ca^2+^]_i_ increase. **F** Average wave trajectories of oscillation ends_50_ (red) with respect to oscillation starts_50_ (blue) for a single oscillation from 6 different islets of Langerhans. Numbers indicate angles in degrees. Note that the wave-fronts of ends_50_ spread in roughly the same direction as the wave-fronts of starts_50_ in most cases. Sampling rate was 50 Hz at a spatial resolution of 256x64 pixels.

To establish a common reference point for the various types of data gained in this study, we first extended our previous work on [Ca^2+^]_i_ waves employing confocal imaging of OGB-1 loaded acute pancreas tissue slices [22]. During the plateau phase of the response to stimulation with 12 mM glucose, beta cells exhibited repetitive oscillatory increases in [Ca^2+^]_i_ that were spreading over the optical plane of the islet of Langerhans as described previously (Figure 2) [22]. To use a robust measure of the onset and end of an oscillation applicable to both [Ca^2+^]_i_ and much noisier membrane potential imaging data, we determined the duration of an oscillation at its half-maximal amplitude. The limits of this interval were used as the start (start_50_) and end (end_50_) of a given oscillation. Since waves of [Ca^2+^]_i_ oscillations often did not follow a linear route, we determined the velocity of the waves by calculating vectorial trajectories describing the average wave paths for the starts_50_ and ends_50_ of a [Ca^2+^]_i_ oscillation (Figure 2E, blue and red dashed line vectors, respectively) which is a more general approach compared with the one used previously [22]. An average trajectory of starts_50_ of a spreading [Ca^2+^]_i_ oscillation was calculated for each of the 6 islets used in this study. Aligning the average directions of starts_50_ of a spreading [Ca^2+^]_i_ oscillation in the different islets at 0° allowed for a comparison of route trajectories of the ends_50_ of [Ca^2+^]_i_ oscillations across different islets (Figure 2F). In four out of six islets, the end_50_ [Ca^2+^]_i_ oscillation direction was within 90° of the start_50_ direction, whereas in the remaining two islets the return of [Ca^2+^]_i_ to the baseline followed a route that was displaced with respect to the start_50_ of [Ca^2+^]_i_ oscillation by more than 90°. In none of the islets the directions of the start_50_ and end_50_ of [Ca^2+^]_i_ oscillations differed by more than 120°.

### Confocal membrane potential imaging

**Figure 3:**
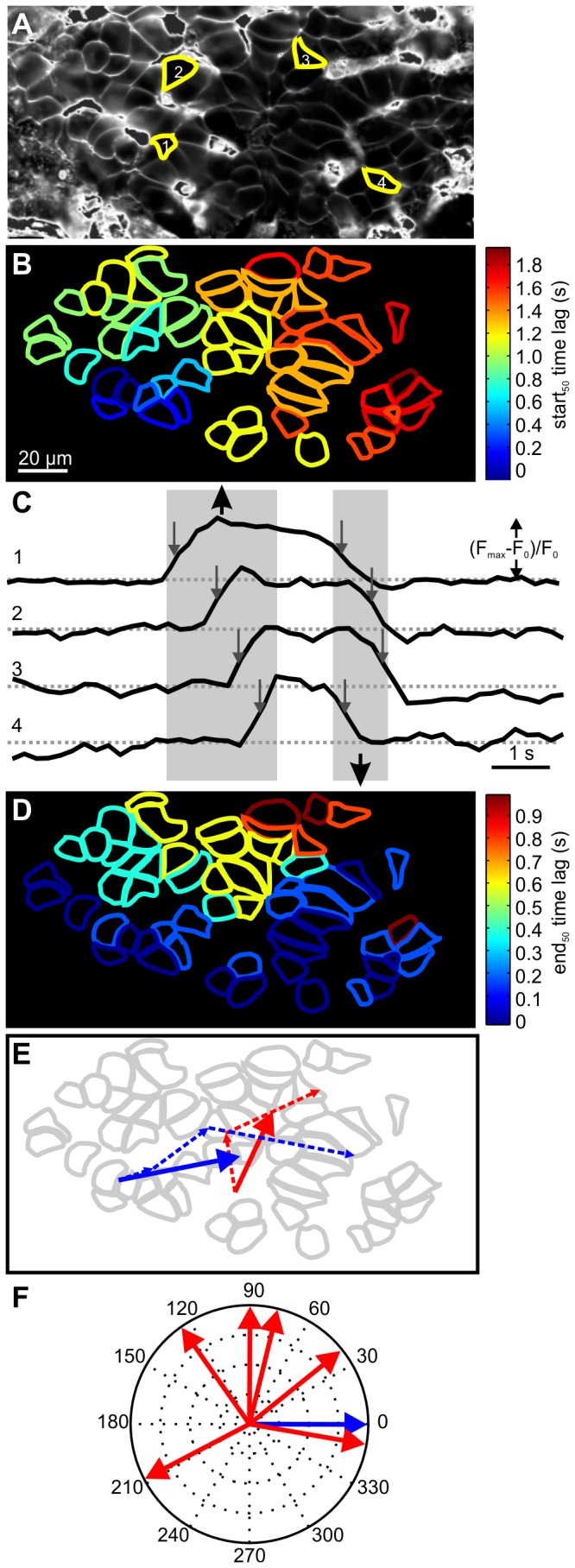
Membrane potential oscillations during stimulation with 12 mM glucose. **A** The VF dye labeled the majority of membranes in this islet of Langerhans from an acute mouse pancreas tissue slice. **B** During stimulation with 12 mM glucose, a wave of depolarization spread across the islet plane from left to right. Time lags between the start_50_ of membrane potential oscillation in the first cell and the rest of the cells are color-coded. **C** The oscillatory behavior during stimulation consisted of depolarizations and repolarizations. A single wave is shown; numbers denote cells´ spatial positions indicated in A. Gray rectangular areas indicate time intervals for depolarizations and repolarizations across different cells. Arrows indicate the duration of oscillations at their half-maximal amplitudes. Y axis represents the normalized fraction of the difference between maximum and plateau baseline fluorescence. **D** In general, the spreading of ends_50_ of oscillations did not take the same route as the starts_50_ of membrane potential oscillations. Time lags are color-coded. **E** Trajectories (dashed lines) and the average trajectory (solid line) for the start_50_ (blue) and end_50_ (red) of the membrane potential oscillation. **D** Repolarization did not follow any clearly predictable pattern in the 6 islets. Shown are angles of average trajectories of oscillation ends_50_ (red) relative to the respective starts_50_ trajectory (blue). Sampling rate was 5 Hz at a spatial resolution of 512x256 pixels.

Next, we assessed the properties of membrane potential oscillations and spreading of membrane potential oscillations at the level of the beta cell functional syncytium in the pancreas tissue slice. This was achieved by using a novel voltage-sensitive dye VoltageFluor (VF) with an enhanced linear fluorescence response per voltage change [28] that enabled simultaneous observation of responses in many cells. VF labeled majority of membranes in islets of Langerhans, enabling determination of location and shape of single cells within a given islet (Figure 3A). For a single cell, the fluorescent response of VF during the plateau phase of the response to 12 mM glucose consisted of oscillations (Figure 3C) with temporal properties resembling the slow waves of the bursting pattern recorded with the patch pipette (Figure 1C). Note that in the VF signal the high frequency spikes superimposed on slow waves that are recorded with the patch pipette are missing from the depolarized phases. For the islet in Figure 3 start_50_ time points of a single membrane potential oscillation were determined for different cells, delays between them color-coded and used to build a spatio-temporal map of the membrane potential oscillation revealing a wave-like spreading of the start_50_ of membrane potential oscillation, starting in the left bottom corner and then spreading toward the right top corner of the frame (Figure 3B). The same analysis was performed for end_50_ time points, showing that repolarization was also spreading over the islet in a wave-like manner. For the islet in Figure 3 the direction of end_50_ of membrane potential oscillation differed from the start_50_ of membrane potential oscillation, spreading from bottom to top. To consistently quantify this phenomenon across different islets, we used an approach similar to the analysis in the [Ca^2+^]_i_ imaging section. The spreading of the membrane potential oscillation was presented as average trajectories for the start_50_ (blue) and end_50_ (red) of the membrane potential oscillations and the directions of the start_50_ trajectories aligned to 0°. In two out of six islets used for this purpose, the average direction of end_50_ of membrane potential oscillation fell within 90° of the average direction of start_50_ of membrane potential oscillation, in two islets end_50_ of membrane potential oscillation progressed in roughly the opposite direction as the start_50_ of membrane potential oscillation (angle > 180°), whereas the remaining two islets displayed intermediate angles of end_50_ of membrane potential oscillation. 

### Comparison of membrane potential and [Ca^2+^]_i_ oscillations during stimulation with glucose

**Figure 4:**
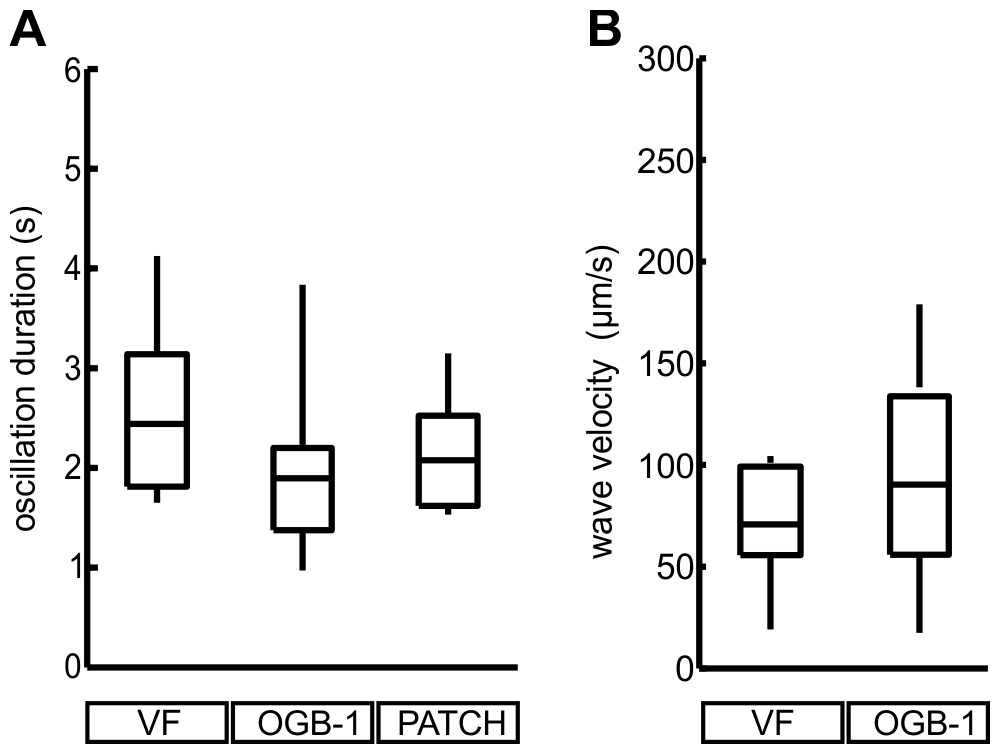
Durations of oscillations and velocities of waves during stimulation with 12 mM glucose are comparable for the [Ca^2+^]_i_ and membrane potential signals. **A** Durations of [Ca^2+^]_i_ oscillations at their half-maximal amplitude in randomly picked cells from 7 islets assessed by OGB-1 (OGB-1, 1^st^ quartile=1.4 s, median=1.9 s, 3^rd^ quartile=2.2 s, n=7 islets), of membrane potential oscillations in randomly picked cells from 7 islets assessed by VF (VF, 1^st^ quartile=1.8 s, median=2.4 s, 3^rd^ quartile=3.2 s, n=7 islets) and of membrane potential oscillations assessed in 7 whole-cell patch-clamped cells (PATCH, 1^st^ quartile=1.6 s, median=2.1 s, 3^rd^ quartile=2.5 s, n=7 islets) are not statistically significantly different (Kruskal-Wallis test). **B** Median membrane potential wave velocities (VF: 1^st^ quartile=55 µm/s, median=69 µm/s, 3^rd^ quartile=97 µm/s, n=7) and [Ca^2+^]_i_ wave velocities (OGB-1: 1^st^ quartile=54 µm/s, median=89 µm/s, 3^rd^ quartile=139 µm/s, n=6) were not statistically significantly different (Mann-Whitney test).

Durations of oscillations in different islets, as determined separately by the confocal imaging of the membrane potential, the [Ca^2+^]_i_, and by the patch-clamp experiments are shown and compared in Figure 4A. Velocities of membrane potential and [Ca^2+^]_i_ oscillation spreading determined by confocal imaging are shown and compared in Figure 4B. The median duration of the membrane potential oscillation (determined as the interval between end_50_ and start_50_) assessed by the VF dye was 2.4 s (Figure 4A, 1^st^ quartile=1.8 s, 3^rd^ quartile=3.2 s, n=7 islets). This value is not statistically significantly different from the median duration of oscillations measured with the patch pipette at a much higher temporal resolution, i.e. 1 kHz (Figure 4A, median=2.1 s, 1^st^ quartile=1.6 s, 3^rd^ quartile=2.5 s, n=7 islets). Additionally, comparing membrane potential oscillation durations with the [Ca^2+^]_i_ oscillations also showed no statistically significant difference (Figure 4A, 1^st^ quartile=1.4 s, median=1.9 s, 3^rd^ quartile=2.2 s, n=7 islets). Finally, the velocities at which the membrane potential and [Ca^2+^]_i_ oscillations were spreading were not statistically significantly different (Figure 4B) (VF: 1^st^ quartile=55 µm/s, median=69 µm/s, 3^rd^ quartile=97 µm/s, n=7; OGB-1: 1^st^ quartile=54 µm/s, median=89 µm/s, 3^rd^ quartile=139 µm/s, n=6). 

### Comparison of membrane potential and [Ca^2+^]_i_ oscillations during stimulation with glucose plus tetraethylammonium

**Figure 5:**
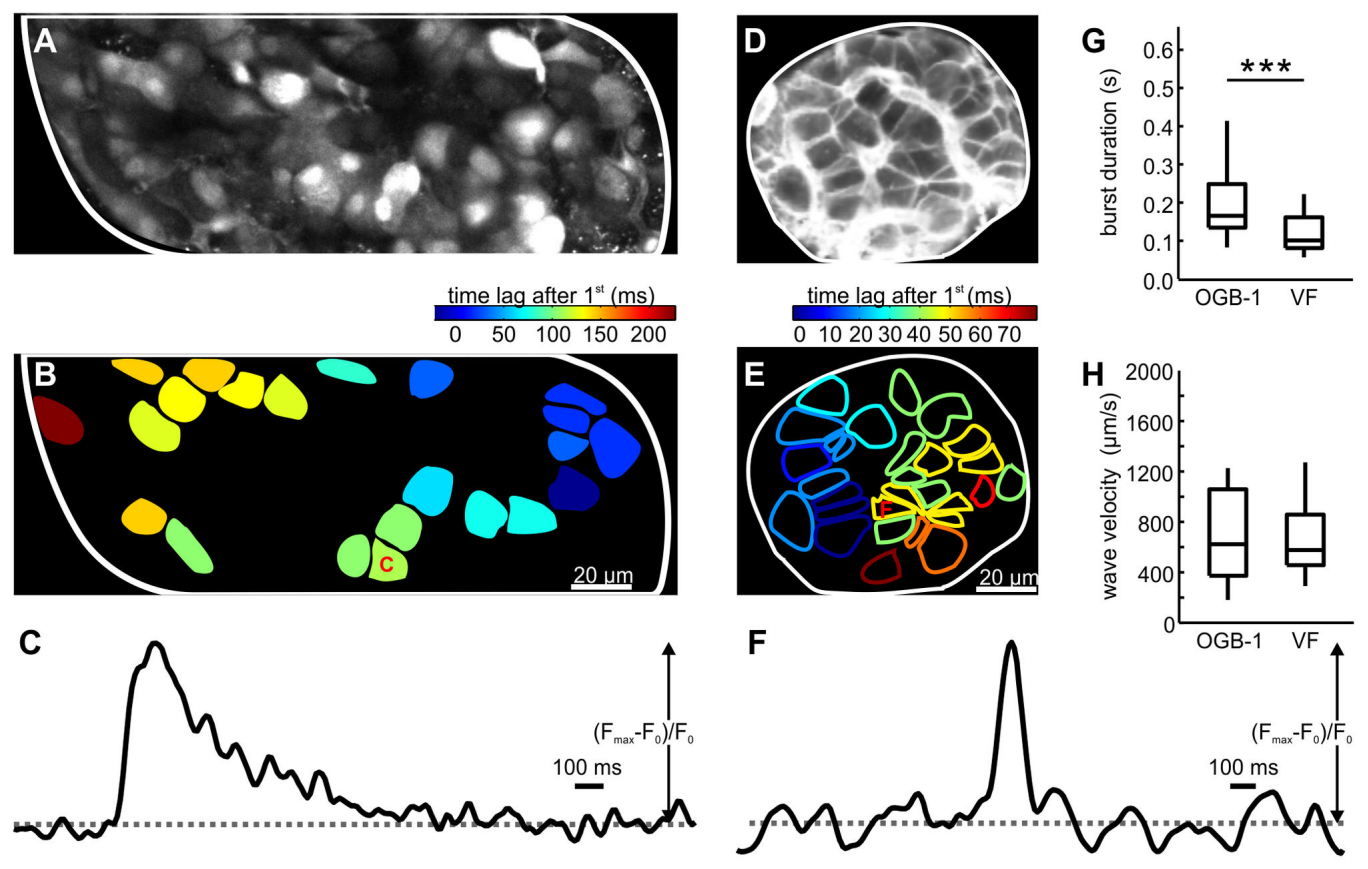
[Ca^2+^]_i_ and membrane potential oscillations during stimulation with 12 mM glucose plus 10 mM tetraethylammonium (TEA). **A** A high resolution image showing loading of cells with the calcium-sensitive dye OGB-1. **B** A color-coded representation of responsive cells from A with respect to time lags between the start_50_ of [Ca^2+^]_i_ oscillation in the first cell and every other cell. **C** Time trace of a [Ca^2+^]_i_ oscillation during stimulation with 12 mM glucose plus 10 mM TEA in the cell indicated in B. **D** A high resolution image showing labeling of cells with the voltage-sensitive dye VF. Note that almost exclusively plasma membranes are labeled. **E** A color-coded representation of responsive cells from D with respect to time lags between the start_50_ of membrane potential oscillation in the first cell and every other cell. **F** Time trace of a membrane potential oscillation during stimulation with 12 mM glucose plus 10 mM TEA in the cell indicated in D. The time scale is the same for C and F. Y axis represents the normalized fraction of the difference between maximum and plateau baseline fluorescence. **G** Durations of [Ca^2+^]_i_ oscillations (OGB-1: 1^st^ quartile=135 ms, median=166 ms, 3^rd^ quartile=248 ms, n=57 cells from three islets) and membrane potential oscillations (VF: 1^st^ quartile=81 ms, median=101 ms, 3^rd^ quartile= 162 ms, n=107 cells from five islets) at their half-maximal amplitude. Asterisks indicate a statistically significant difference (p<0.001, Mann-Whitney rank sum test). **H** Velocities of [Ca^2+^]_i_ waves (OGB-1: 1^st^ quartile=372 µm/s, median=623 µm/s, 3^rd^ quartile=1058 µm/s, n=8 values from three islets) and membrane potential waves (VF: 1^st^ quartile=457 µm/s, median=577 µm/s, 3^rd^ quartile=857 µm/s, n=12 values from five islets) were not statistically significantly different. Resolution was 256 x 64 pixels at 97 Hz for OGB-1 and 256x128 at 99Hz for VF.

For the above oscillation durations, the rather large variability between cells from different islets could mask any potential systematic differences between durations of the membrane potential and [Ca^2+^]_i_ oscillations during stimulation with 12 mM glucose. Thus, to check whether membrane potential and [Ca^2+^]_i_ oscillations possibly display any systematic differences, we took advantage of the well described phenomenon that stimulation of glucose-stimulated beta cells with the potassium channel blocker tetraethylammonium (TEA) at concentrations above 5 mM replaces the characteristic bursting pattern (slow waves with superimposed fast spikes) with continuous spiking consisting of much taller and longer spikes with no underlying slow waves [34,35]. We reckoned that a much larger amplitude of membrane potential oscillations could effectively unmask possibly slower [Ca^2+^]_i_ kinetics. Additionally, since the currently achievable combination of imaging speed and voltage indicator properties did not enable us to resolve individual fast spikes superimposed on slow waves during stimulation with glucose, stimulation with TEA in addition to glucose enabled us to characterize the taller and longer spikes present during stimulation with stimulatory glucose plus TEA and their degree of synchronization in different cells. Figure 5 summarizes the properties of membrane potential and [Ca^2+^]_i_ oscillations in islets of Langerhans during stimulation with 12 mM glucose plus 10 mM TEA. We used the generic term *oscillations* to describe the membrane potential changes detectable by VF and produced by slow waves with superimposed spikes in case of stimulation with glucose only, as well as to describe the membrane potential changes detectable by VF and produced by large amplitude regular spikes with no underlying slow oscillations in case of stimulation with glucose plus TEA. Similarly to stimulation with glucose only, both membrane potential and [Ca^2+^]_i_ oscillations spread across the focal plane in a wave-like manner. However, the [Ca^2+^]_i_ signal differed markedly from the membrane potential signal in terms of its kinetics, the [Ca^2+^]_i_ return to the baseline being markedly prolonged compared with the membrane potential signal, which is reflected in significantly longer durations of [Ca^2+^]_i_ oscillations compared with the membrane potential oscillations (Figure 5G, OGB-1: 1^st^ quartile=135 ms, median=166 ms, 3^rd^ quartile= 248 ms, n=57 cells; VF: 1^st^ quartile=81 ms, median=101 ms, 3^rd^ quartile= 162 ms, n=107 cells). Interestingly, TEA also increased the speed at which the membrane potential and [Ca^2+^]_i_ waves spread over the islet by almost ten-fold (Figure 5H, OGB-1: 1^st^ quartile=372 µm/s, median=623 µm/s, 3^rd^ quartile=1058 µm/s, n=8; VF: 1^st^ quartile=457 µm/s, median=577 µm/s, 3^rd^ quartile=857 µm/s, n=12; for comparison, median velocities during stimulation with glucose only were 69 µm/s for VF and 89 µm/s for OGB-1 signal). For comparison of velocities at which the membrane potential and the [Ca^2+^]_i_ wave spread in islets during stimulation with glucose with and without TEA see also Figure S2.

### Simultaneous measurement of membrane potential and [Ca^2+^]_i_ oscillations during stimulation with glucose

**Figure 6:**
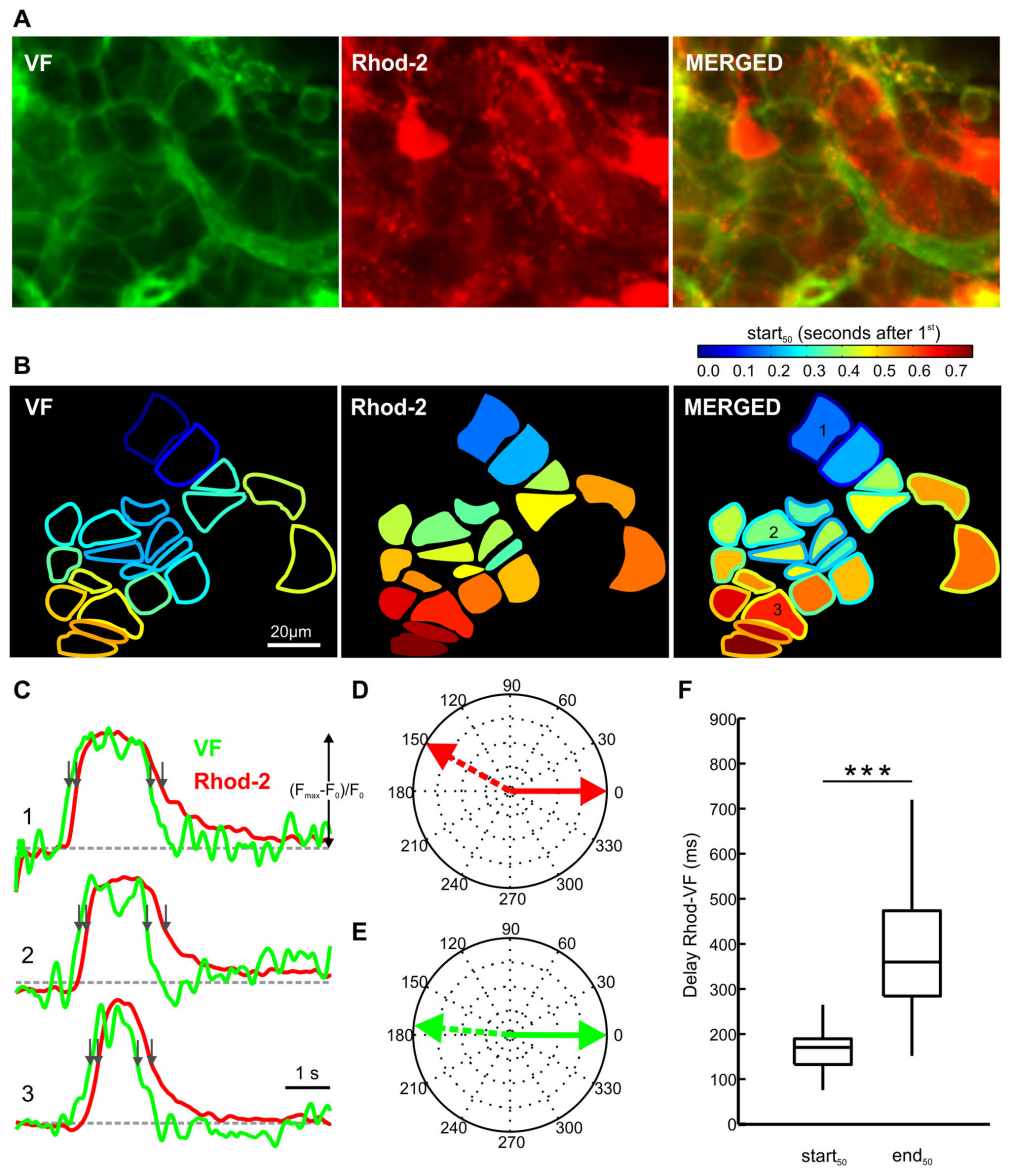
Simultaneous recording of [Ca^2+^]_i_ and membrane potential oscillations during stimulation with 12 mM glucose. **A** VF (left panel) stained mostly cell membranes while Rhod-2 (middle panel) stained cell cytoplasms, without any significant co-localization of the two dyes in the active regions (right panel; see also Figure S4). **B** A color-coded representation of selected responsive cells with respect to the time lag between the oscillation start_50_ of the first cell and every other cell for the VF signal (left panel), Rhod-2 signal (middle panel) and merge of the two (right panel). The outline stands for the VF signal and the interior for Rhod-2. Both membrane potential and [Ca^2+^]_i_ wave roughly spread from top to bottom of the focal plane. Note that in any given cell, the membrane signal precedes (colder color) the [Ca^2+^]_i_ signal, indicating that depolarization precedes the increase in [Ca^2+^]_i_. **C** Time traces of the VF and Rhod-2 signals from three cells indicated in the right panel of B show a wave-like spreading of both signals. In all cells, the VF signal clearly preceded the Rhod-2 signal. Arrows indicate the widths of oscillations at their half-maximal amplitude (see Methods). Y axis represents the normalized fraction of the difference between maximum and plateau baseline fluorescence. **D** Average trajectory for [Ca^2+^]_i_ oscillation starts_50_ (solid line) and ends_50_ (dashed line). **E** Average trajectory for the membrane potential oscillation starts_50_ (solid line) and ends_50_ (dashed line). In D and E, numbers indicate angles in degrees. **F** Time lag between ends_50_ (1^st^ quartile=284 ms, median= 360 ms, 3^rd^ quartile=473 ms) was significantly larger than the time lag between starts_50_ (1^st^ quartile=133 ms, median=170 ms, 3^rd^ quartile=189 ms, n=21 cells) of the Rhod-2 and VF signals. Asterisks indicate p<0.001 (Mann-Whitney test). Resolution was 256x128 pixels at 52 Hz.

To unambiguously resolve whether any of the two signals precedes the other at the level of individual cells, we took up the task of simultaneously staining beta cells with calcium- and voltage-sensitive dyes. We successfully recorded from two islets the simultaneous response of the membrane potential and [Ca^2+^]_i_ reporter dyes during stimulation with 12 mM glucose (Figure 6). VF loaded mostly the cell membranes whilst the [Ca^2+^]_i_ was recorded using the Rhod-2 dye instead of OGB-1 due to its spectral properties that enabled excitation of both dyes using two laser lines and spectrally discriminating the two emitted signals (for details see Figure S4). As expected, the membrane potential oscillation spread in a wave-like manner from top to bottom in the Figure 6, with [Ca^2+^]_i_ oscillation following in the same direction, a property that could not be conclusively resolved by recording membrane potential and [Ca^2+^]_i_ oscillations separately. Importantly, the start_50_ of the membrane potential oscillations preceded the start_50_ of the [Ca^2+^]_i_ oscillations by a median of 170 ms (Figure 6F, 1^st^ quartile=133 ms, 3^rd^ quartile=189 ms, n=21 cells). The described time delay is clearly visible in time traces in Figure 6C. This delay can be visualized in a merged spatio-temporal map of the two signals, with time delays color-coded using the same scale for both signals (Figure 6B, rightmost panel). In each cell, the membrane is pseudo-colored with a cooler color than its respective interior, clearly indicating that cell membrane depolarizes first, which is followed by a detectable increase in [Ca^2+^]_i_. The median time lag between end_50_ of the membrane potential and [Ca^2+^]_i_ oscillations was 360 ms (Figure 6F, 1^st^ quartile=284 ms, 3^rd^ quartile=473 ms).

### Simultaneous measurement of membrane potential and [Ca^2+^]_i_ oscillations during stimulation with glucose plus tetraethylammonium

**Figure 7:**
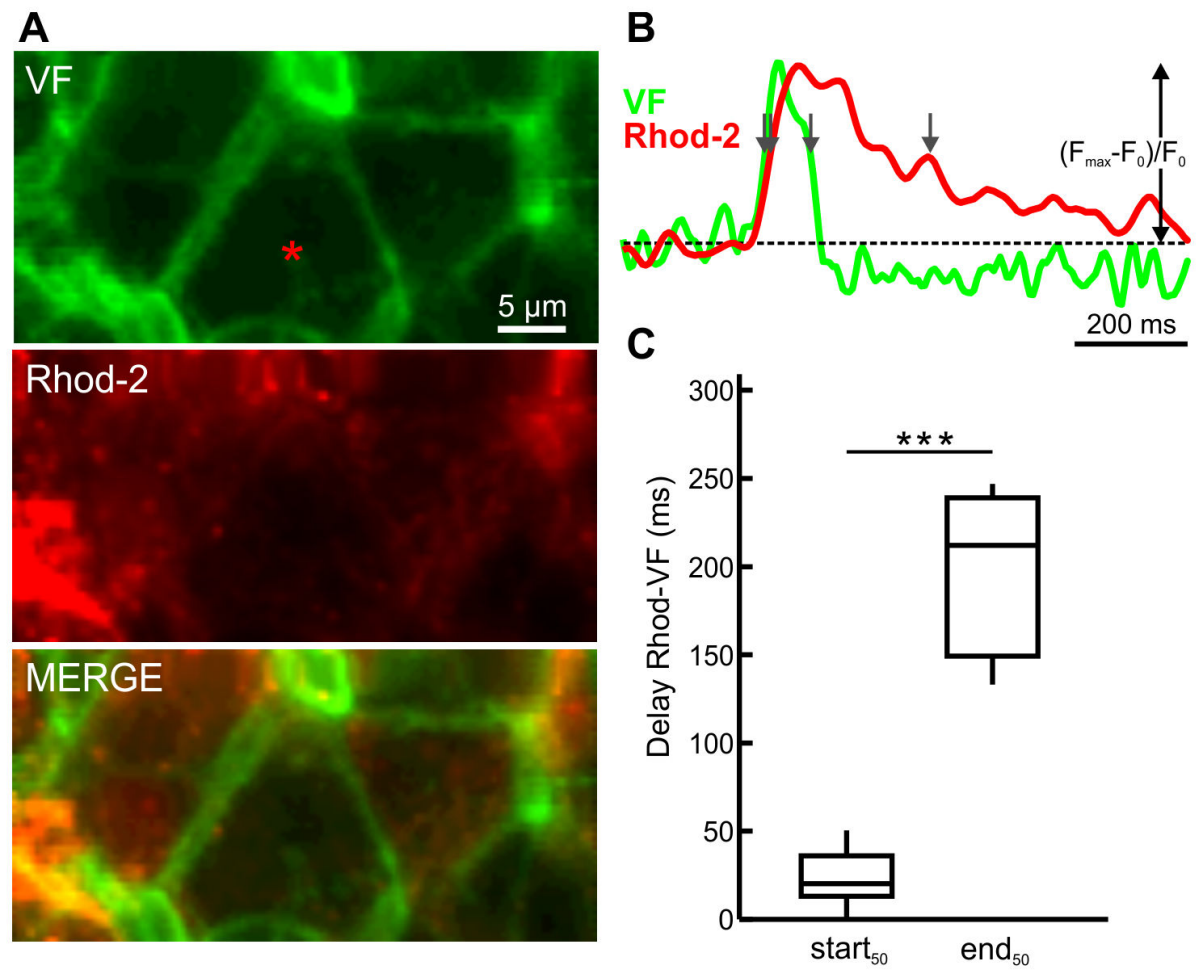
Simultaneous recording of [Ca^2+^]_i_ and membrane potential oscillations during stimulation with 12 mM glucose plus 10 mM tetraethylammonium (TEA). **A** The voltage sensitive dye VF (upper panel) stained mostly cell membranes, whereas the calcium indicator Rhod-2 (middle panel) stained cytoplasms. For the active cells no significant co-localization was observed (lower panel). **B** Oscillation in the cell indicated with red asterisk in A during stimulation with 12 mM glucose plus 10 mM TEA. Arrows indicate the widths of oscillations at their half-maximal amplitude. Y axis represents the normalized fraction of the difference between maximum and plateau baseline fluorescence. **C** The delay between start_50_ of the Rhod-2 and VF signals (1^st^ quartile=13 ms, median=20 ms, 3^rd^ quartile=36 ms, n=11 cells from 4 islets) was statistically significantly shorter than the delay between end_50_ of the Rhod-2 and VF signals (1^st^ quartile=150 ms, median=212 ms, 3^rd^ quartile=239 ms, n=11 cells from 4 islets). Asterisks indicate p<0.001 (Mann-Whitney test). Resolution was 128x64 pixels at 170 Hz.

Finally, we wanted to explore how coupling between membrane potential and [Ca^2+^]_i_ oscillations at the level of individual cells changes when 12 mM glucose plus 10 mM TEA are used for stimulation. To clarify this, we double-stained four islets with the membrane potential and [Ca^2+^]_i_ reporter dyes and recorded their responses during stimulation. With this protocol, the differences in the kinetics of both signals became even more obvious. The VF signal showed a very fast upstroke and return to the baseline, whereas the Rhod-2 signal showed slower upstroke of the signal and its return to the baseline was slower by several tens of milliseconds. The time lag between the start_50_ of the membrane potential and of the [Ca^2+^]_i_ oscillation was significantly decreased compared with oscillations in glucose only (Figure 7C, 1^st^ quartile=13 ms, median=20 ms, 3^rd^ quartile=36 ms, n=11 cells from 4 islets). Conversely, the time lag for the ends_50_ was decreased to a smaller extent (Figure 7C, 1^st^ quartile=150 ms, median=212 ms, 3^rd^ quartile=239 ms, n=11 cells from 4 islets) compared with conditions without TEA.. For comparison of parameters during stimulation with glucose and glucose plus TEA, see Figure S3. 

## Discussion

A qualitative description of the extent of coupling between membrane potential and [Ca^2+^]_i_ oscillations using the patch-clamp technique and CCD camera imaging has been previously attempted on isolated islets [17,19]. This approach however has some major drawbacks. First, the islet core remained largely inaccessible to the calcium-sensitive dye in isolated islets. Taking into account that the beta cells predominantly populate the islet core, this shortcoming is critical. Recently we reported that the method of pancreatic tissue slice readily allows access to the islet core allowing the experimenter to record from hundreds of beta cells simultaneously [22]. Secondly, the highest temporal resolution in the CCD imaging experiments was between 0.3 - 3 Hz [17,19]. At such resolution, the change in membrane potential seems to accompany the [Ca^2+^]_i_ oscillation as both processes seem time locked. In our hands, 100 ms exposure of the sample to the fluorescent light has been sufficient to image the OGB-1 signal; however, using this exposure period with a frequency higher than 3Hz completely abolished the membrane potential oscillatory activity in the patch-clamped cell thus preventing higher temporal resolution recordings using the CCD camera. Finally, the classical electrophysiological approach of recording membrane potential has the inherent limitation that only a few cells at most can be recorded per islet in parallel. This strongly limits the amount of available data that would reliably describe the electrical network of beta cells and therefore describe the coupling of the membrane potential and [Ca^2+^]_i_ on a population level. 

Recently, a novel family of voltage-sensitive dyes named VoltageFluor (VF) with increased sensitivity to voltage changes compared with older generation voltage-sensitive dyes was reported [28]. This property is of crucial importance for a beta cell physiologist, since in beta cells, the native membrane potential excursions during stimulation with physiological secretagogues occur in the range of several tens of millivolts at most, which is hardly detectable by using less sensitive classical voltage-sensitive dyes. 

The VF signal recorded from islets of Langerhans in acute pancreas tissue slices during stimulation with glucose showed the characteristic oscillatory pattern, very similar in period to the slower carrying component of the one observed with the patch pipette (i.e. bursts) during the plateau phase of the response to 12 mM glucose (Figures 1 and 3). High frequency spikes occurring during the bursts, detectable with electrophysiological methods, were missing in the VF signal. Since the duration of individual spike is 100 ms or shorter, recordings with sampling rate < 50 Hz are too slow to allow detection of individual spikes. Increasing sampling rate (99 Hz, data not shown) produced signal to noise ratio that did not allow for spike detection using standard analysis. Since the major focus of our current study were the detection and characterization of spreading of slow waves or bursts as a whole as well as the comparison of membrane potential and [Ca^2+^]_i_ dynamics, we did not further investigate this issue.

Time lags between start_50_ of oscillations formed a specific spatio-temporal activation map that for the first time clearly revealed a wave-like spreading of depolarization over the optical plane of the islet. Comparing properties of membrane potential dynamics with the ones of [Ca^2+^]_i_ dynamics showed that membrane potential oscillations were of shorter duration than [Ca^2+^]_i_ oscillations during stimulation with 12 mM glucose at the single cell level. The durations of membrane potential and [Ca^2+^]_i_ oscillations assessed non-simultaneously in different islets did not differ from the durations of bursts, assessed by using the patch pipette, a method that has a several-fold higher temporal resolution compared with imaging approaches. The median velocity at which the depolarization spread over the islets was 69 µm/s, a value very similar to the velocity of [Ca^2+^]_i_ oscillations measured in tissue slices in this study (Figure 4B) and previously [22], as well as to the velocity of membrane potential oscillations estimated in paired-electrode studies [24] and [Ca^2+^]_i_ oscillations detected in cultured isolated islets [18]. 

Palti et al. [25] recorded the electrical activity of islets with extracellular electrodes positioned at islet surface in a fashion similar to the recording of brain waves, i.e. electroencephalogram, and even named it electroisletgram. From a theoretical perspective, such electrical activity of beta cells can only be detected if (i) islets contain functional pacemaker cells from which a wave of electrical depolarization propagates and (ii) these cells are located in cell layers near the islet surface [36]. Only in this case, measurable systematic differences in electric potential exist between the two electrodes. If the membrane potential oscillations of all cells were completely synchronized or if the depolarization wave spread from the center, no measurable differences in electric potential at the two electrodes would exist. In our study, we confirmed that there is a directed propagation of depolarization supporting the electroisletgram findings. Namely, in all of the islets a clear depolarization wave was detected and it always started at islet periphery. 

For both membrane potential and [Ca^2+^]_i_ signal, the return to baseline, assessed by the end_50_ parameter, also formed a wave spreading over the focal plane of the islet (Figures 2 and 3). We constructed trajectories for each oscillation end_50_, using them as descriptors for the pathway of the end_50_ wave-fronts. Surprisingly, the end_50_ spreading directions of the membrane potential and [Ca^2+^]_i_ oscillations could not be unequivocally predicted from the start_50_ directions of the membrane potential or [Ca^2+^]_i_ oscillations (Figures 2F and 3F). In contrast to myocardium, in islets of Langerhans, the cells that depolarize first might repolarize first or last, suggesting much less evolutionary pressure on islets with changing directions of depolarization. In our view, the relationship between velocity of wave propagation (100 μm/s), oscillation duration (2-3 s) and the characteristic islet size (100-300 μm) strongly suggests that the simultaneous activity of all cells in the form of synchronous oscillations during the sustained phase seems to offer some evolutionary advantage, and this behavior will be present even if the direction of waves changes with time. The discrepancy in start_50_ and end_50_ directions of oscillations also explains the variability of oscillation durations across different cells from the same islet observed in this and our previous study [22]. 

In the next step we aimed at determining differences in kinetics of the membrane potential and [Ca^2+^]_i_ oscillations. Addition of > 5mM tetraethylammonium (TEA) to a stimulatory concentration of glucose replaces the bursting pattern (i.e. slow waves with superimposed spikes) with continuous spiking consisting of much taller and longer spikes only (i.e. with no underlying slow waves) that reach from the threshold of slow waves to positive values and are of approximately double the amplitude than slow waves with superimposed spikes combined. The duration of these spikes is an order of magnitude shorter than the duration of slow waves with superimposed spikes (which last several seconds) but several times longer than the duration of individual spikes superimposed on the slow waves [34,35]. It seemed logical to take advantage of the membrane potential spikes with large amplitudes, steep slope, and a shorter duration compared with the burst to look for the presence of significant delays in the [Ca^2+^]_i_ dynamics under this conditions and to see whether waves would still be detectable and their velocity of spreading would increase. The physiological background underlying the effect of TEA is beyond the scope of this study [37-39]. Using the VF dye, we were able to record the expected effect of TEA: individual membrane potential oscillations with a duration of 100 ms, a value comparable to the one recorded in sharp electrode experiments [34]. What is more, plotting the spatio-temporal map of the oscillation start_50_ showed that during stimulation with glucose plus TEA oscillations spread over the islet in the form of a wave (Figure 5), a property largely overlooked before due to technical limitations. Interestingly, the median velocity at which the membrane potential oscillation propagated over the focal plane of the islet was 577 µm/s; the velocity increased almost 10-fold with respect to the velocity recorded during stimulation with glucose only (Figure 5F and Figures S2-S3). This increase in velocity corroborates and explains the previous finding that stimulation with glucose plus TEA decreases time lags between membrane potential oscillations of the impaled cell and the average [Ca^2+^]_i_ oscillations from the whole islet [17]. During stimulation with glucose plus TEA, velocities of [Ca^2+^]_i_ and membrane potential waves did not differ significantly. On average, [Ca^2+^]_i_ oscillations lasted 37% longer than membrane potential oscillations, measured at the oscillation half-maximal amplitude (Figure 5G). The latter was a consequence of a markedly prolonged return of [Ca^2+^]_i_ to the baseline compared with the membrane repolarization. The latter is in accordance with previous findings that the decrease in [Ca^2+^]_i_ upon termination of a burst is prolonged due to leakage of Ca^2+^ from the endoplasmic reticulum, delaying the emptying of the cytosolic pool of Ca^2+^ by membrane pumps [40]. 

So far, studying membrane potential oscillations in beta cells has been constrained by the use of slow oxonol probes and cell lines for which it is not clearly established to what extent they represent valid models of beta cells [41-44]. We therefore set out to combine a novel voltage sensitive dye with superior sensitivity to voltage changes and a rapid temporal response with our *in situ* tissue slice preparation. We successfully loaded the beta cells within tissue slices with the VF and the Rhod-2 dye, the latter being a [Ca^2+^]_i_ reporter dye chosen in our double-staining experiments due to its spectral properties. Although a considerable spectral difference between the two dyes exists, special care was taken to assure that we were not looking at an artifact of emission cross-talk of the Rhod-2 dye into the VF channel and *vice versa* (Figure S4). Using the double staining approach during stimulation with 12 mM glucose, we were able to simultaneously record membrane potential and [Ca^2+^]_i_ oscillations at a resolution of single cells. Using the double staining approach, we clearly established that during stimulation with 12 mM glucose, the membrane potential and [Ca^2+^]_i_ wave follow the same path and that the former precedes the latter by a median of 170 ms, a property that could be predicted, but not conclusively resolved from recording membrane potential and [Ca^2+^]_i_ changes separately. Additionally, an implicit assumption of the canonical model of beta cell stimulus-secretion coupling is that the activity of individual beta cells is synchronous, without stating clearly to what extent [45,46], or that depolarization propagates from cell to cell electrotonically [7]. However, to date this assumption, as far as it concerns membrane potential changes and not some surrogate marker such as [Ca^2+^]_i_ [22], has not been substantiated directly on a large number of coupled cells from all layers of an islet. Moreover, the end_50_ of [Ca^2+^]_i_ oscillations followed the same route as the end_50_ of the membrane potential oscillation. 

A likely explanation for the behavior during depolarization is that a critical depolarization is needed to trigger Ca^2+^ influx into the cell cytoplasm, since the increase in [Ca^2+^]_i_ starts when membrane potential is still rising. This initial depolarization probably stems from a depolarizing current injected into a given cell from its already active neighbors. This current could be mediated by Ca^2+^ or some other cation. In the former case, it is conceivable that a very small amount of Ca^2+^ ions, undetectable by the fluorescent probe, suffices to depolarize the plasma membrane with small capacity and high input resistance. In the latter case, the ions that depolarize the plasma membrane are not detectable by the fluorescent probe. As to the repolarization phase of the membrane potential oscillation, in our opinion the most likely explanation is that the repolarizing stimulus is efficiently propagated from cell to cell and it appears that the cell to cell heterogeneity in activity of the [Ca^2+^]_i_ handling machinery is not large enough as to produce a significant dissociation between the spatio-temporal properties of the repolarizing membrane potential changes and the return of [Ca^2+^]_i_ back to the baseline. Additionally, time lags could be exaggerated and in the worst case even produced by properties of the Rhod-2 dye. Our study did not enable measuring the absolute value of [Ca^2+^]_i_. However, due to the previously published values of [Ca^2+^]_i_ during the plateau phase with superimposed oscillations that are believed to occur in the range 100-300 nM [19,47], together with the sensitivity of Rhod-2 for [Ca^2+^]_i_ and its kinetic properties [48], we believe it is highly unlikely that the observed delay is a consequence of dye properties. 

To test our hypothesis that the rate of initial depolarization determines when [Ca^2+^]_i_ will start to rise, we employed stimulation with glucose plus TEA in the double-stained slices. Due to the higher amplitude and faster membrane potential changes in this case, combined with an increased input resistance (decreased membrane conductance due to blockade of K^+^ channels), the electrotonic current to neighboring cells should be larger and thus more efficient at depolarizing the neighboring cell to the threshold for triggering its own action potential. Moreover, once the larger amplitude and faster membrane potential change is triggered, [Ca^2+^]_i_ is expected to follow the membrane potential change more rapidly. In addition to the several-fold increased velocity of the membrane potential and [Ca^2+^]_i_ oscillation spread established already in the single-staining experiments, our double-staining experiments revealed that during stimulation with glucose plus TEA, detectable time lags between the onsets of the membrane potential and [Ca^2+^]_i_ oscillations indeed decreased at the employed sampling frequency. In contrast, upon repolarization, the delay in [Ca^2+^]_i_ return to baseline was decreased to smaller extent under these conditions indicating that the decrease in [Ca^2+^]_i_ upon termination of electrical activity is limited by the activity of the [Ca^2+^]_i_ handling machinery. 

## Conclusions

In the present paper, we demonstrated that during stimulation with glucose oscillatory membrane potential changes spread over the islet in a wave-like manner. The duration of membrane potential oscillations was shown to be shorter than the duration of [Ca^2+^]_i_ oscillations, while the velocity at which depolarization spread over the islet was comparable to the one for [Ca^2+^]_i_ waves. For beta cells in their native environment, we reported for the first time simultaneous records of membrane potential and [Ca^2+^]_i_ oscillations during stimulation with glucose at cellular resolution. The membrane potential oscillations were phase-locked to the [Ca^2+^]_i_ oscillations, with the former preceding the later by > 100 ms at the oscillation starts and by > 300 ms at the oscillation ends. The potassium channel blocker tetraethylammonium (TEA) increased the spreading velocity of both membrane potential and [Ca^2+^]_i_ oscillations by several-fold and emphasized differences in the kinetics of membrane potential and [Ca^2+^]_i_ changes. The delay between the membrane potential and [Ca^2+^]_i_ oscillations was decreased to < 50 ms at start_50_ and to < 250 ms at ends_50_. Observed kinetic differences suggest that an in-depth analysis of the electrotonic spread between individual beta cells and of molecular determinants of intracellular calcium homeostasis has the potential to further elucidate the function of the beta cell syncytium in health and disease.

In our opinion, combining voltage- and calcium-sensitive dyes with high resolution confocal imaging in acute tissue slices provides a powerful tool for studying the beta cell syncytium and similar cellular syncytia. Our work upgrades our understanding of how membrane potential and [Ca^2+^]_i_ signaling are coupled at the level of individual cells and how they are integrated at the level of an islet, enabling individual cells to function as a unit. 

## Supporting Information

Figure S1 **Membrane potential oscillations upon stimulation with 12 mM glucose.** **A** Fluorescence trace of the voltage-sensitive dye VF measured with confocal imaging. Cell location within islet of Langerhans is marked in B. Numbers denote glucose concentration in mM. **B** Cell membranes in a representative islet of Langerhans are labeled with the voltage-sensitive dye VF. **C** Membrane potential oscillations indicated in panel A with the gray rectangular area are shown in detail. Y axis represents the normalized fraction of the difference between maximum and plateau baseline fluorescence. Sampling rate 1Hz at 512 x 256 pixels (from which 246 x 256 frame is shown).
(TIF)

Figure S2 **Velocities of [Ca]_i_ and membrane potential waves during stimulation with 12 mM glucose and 12 mM glucose plus 10 mM TEA.** Distance vs. time is plotted for the calculated trajectories during stimulation with 12 mM glucose (blue) and 12 mM glucose plus 10 mM TEA (red), assessed using the VF (circles) and the OGB-1 (diamonds) dye. The respective linear regression lines are drawn for the VF (solid line) and OGB-1 (dashed line) data during stimulation with glucose only (blue) and with glucose plus TEA (red). Note that the slopes of the regression lines directly represent the wave velocities: 188 and 106 µm/s for the VF and OGB trajectories during glucose only stimulations (27 data from 7 islets and 16 data from 6 islets, respectively) and 769 and 1031 µm/s for the VF and OGB trajectories during glucose plus TEA stimulation (12 data from 5 islets and 8 data from 3 islets, respectively).
(TIF)

Figure S3 **Delays between the Rhod-2 and VF signals of starts_50_ and ends_50_ during stimulation with glucose and glucose plus TEA.** **A** During stimulation with 12 mM glucose the delay between starts_50_ of the Rhod-2 and VF signals (1^st^ quartile=133 ms, median=170 ms, 3^rd^ quartile=189 ms, n=21 cells) is statistically significantly longer than during stimulation with 12 mM glucose plus 10 mM TEA (1^st^ quartile=13 ms, median=20 ms, 3^rd^ quartile=36 ms, n=11 cells). Asterisks indicate p<0.001 (Mann Whitney test). **B** During stimulation with 12 mM glucose the delay between ends_50_ of the Rhod-2 and VF signals (1^st^ quartile=284 ms, median=360 ms, 3^rd^ quartile=473 ms, n=21 cells) is statistically significantly longer than during stimulation with 12 mM glucose plus 10 mM TEA (1^st^ quartile=150 ms, median=212 ms, 3^rd^ quartile=239 ms, n=11). Asterisk indicates p<0.05 (Mann Whitney test).
(TIF)

Figure S4 **Experimental setup for simultaneous recording of membrane potential (using VF dye) and [Ca^2+^]_i_ (using Rhod-2 dye).** **A** VF (left panel) stained mostly cellular membranes, whereas Rhod-2 stained the cytoplasms (middle panel). For the active cells, no significant co-localization was observed (right panel). **B-D** Outlines of cells in A (left). Time traces for the VF and Rhod-2 are shown; the respective regions of interest are indicated on the left. Y axis represents the normalized fraction of the difference between maximum and plateau baseline fluorescence. **E** Absorption spectra for the VF and Rhod-2 dyes, indicated are the two laser lines used to excite the dyes. **F** Emission spectra for the VF and Rhod-2 dyes, indicated are the wavelength intervals in which emitted light was detected. Resolution was 128x64 pixels at 170 Hz.
(TIF)

Figure S5 **Comparison of [Ca^2+^]_i_ dynamics measured with two different calcium indicators, OGB-1 and Rhod-2.** **A** Slices were loaded with a loading mixture containing both OGB-1 AM and Rhod-2 AM. Indicated is a region of interest that is analyzed in C-F. **B** Excitation (broken line) and emission (solid line) spectra of OGB-1 (green) and Rhod-2 (red). Vertical broken lines indicate the 488 nm laser light exciting both dyes and the 561 nm laser light exciting Rhod-2 only. Detector 1 was set to measure OGB-1 signal only (0.4 % of the Rhod-2 emission is detected) whereas the detector 2 captured mostly Rhod-2 and partly OGB-1 emission (20 % of the total OGB-1 emission is detected with this detector). **C** 12 mM glucose elicited [Ca^2+^]_i_ oscillations superimposed on the plateau phase of the response. Both detectors measured equal shapes of the seven [Ca^2+^]_i_ oscillations. Note that the red signal is several times larger than the green signal at equal detector gains. In this and all other panels, signals from detector 1 and 2 are depicted in green and red, respectively. **D** A detailed representation of a single oscillation from C. [Ca^2+^]_i_ dynamics of the green and red signals are identical. **E** The oscillation onset from panel D is shown in detail. For both the green and the red signals the starts_50_ of [Ca^2+^]_i_ oscillations were determined at their half-maximal amplitude (indicated by arrows). Original (thin lines) and filtered data (thick lines) are shown. **F** End of the oscillation from panel D is shown in detail. Ends_50_ of [Ca^2+^]_i_ oscillations were determined for the red and green signals at their half-maximal amplitude (indicated by arrows). Original (thin lines) and filtered data (thick lines) are shown. **G** Differences in starts_50_ and ends_50_ of [Ca^2+^]_i_ oscillations of the red and green signals for the 7 oscillations in 9 cells. The difference between the two signals was not statistically significantly different from zero for starts_50_ and ends_50_ (p>0.05).
(TIF)

Figure S6 **Simultaneous recording of membrane potential change with the VF dye and with the patch-clamp technique.** **A** The VF dye loaded many cells in this islet of Langerhans. White lines indicate location of the patch-clamp pipette. **B** A scheme indicating active cells. The patch pipette is in contact with the cell from which the membrane potential was recorded with the whole-cell patch-clamp technique. Its neighboring cells from which the membrane potential was measured as VF fluorescence are marked with numbers. **C** In the patched-clamped cell the membrane potential depolarization was followed by superimposed spikes (lower trace marked with PATCH V_m_). In addition to the raw signal, a smoothed signal of the membrane potential change is shown (thicker solid line). In the neighboring cells the VF fluorescence transiently increased to a new plateau value (traces marked with VF V_m_). Note the equal timing and shape of the fluorescence signal compared to the smoothed signal recorded with the patch-clamp. The high frequency spikes are missing from the fluorescence signal due to sampling rate of the VF signal (5 Hz). Sampling rate for the patch-clamp was 1000 Hz. Traces represent the normalized fraction of the difference between maximum and plateau baseline fluorescence. The VF fluorescence was captured using the CCD camera at 256x256 pixels.
(TIF)

Text S1 **Substantiation of the Rhod-2 as indicator of cytosolic [Ca^2+^].**
(DOCX)
