# Supplementary figures and images for: The Relationship between Membrane Potential and Calcium Dynamics in Glucose-Stimulated Beta Cell Syncytium in Acute Mouse Pancreas Tissue Slices
Source: PLoS One. 2013 Dec 6;8(12):e82374. doi: 10.1371/journal.pone.0082374 (PMC3855743; doi:10.1371/journal.pone.0082374)

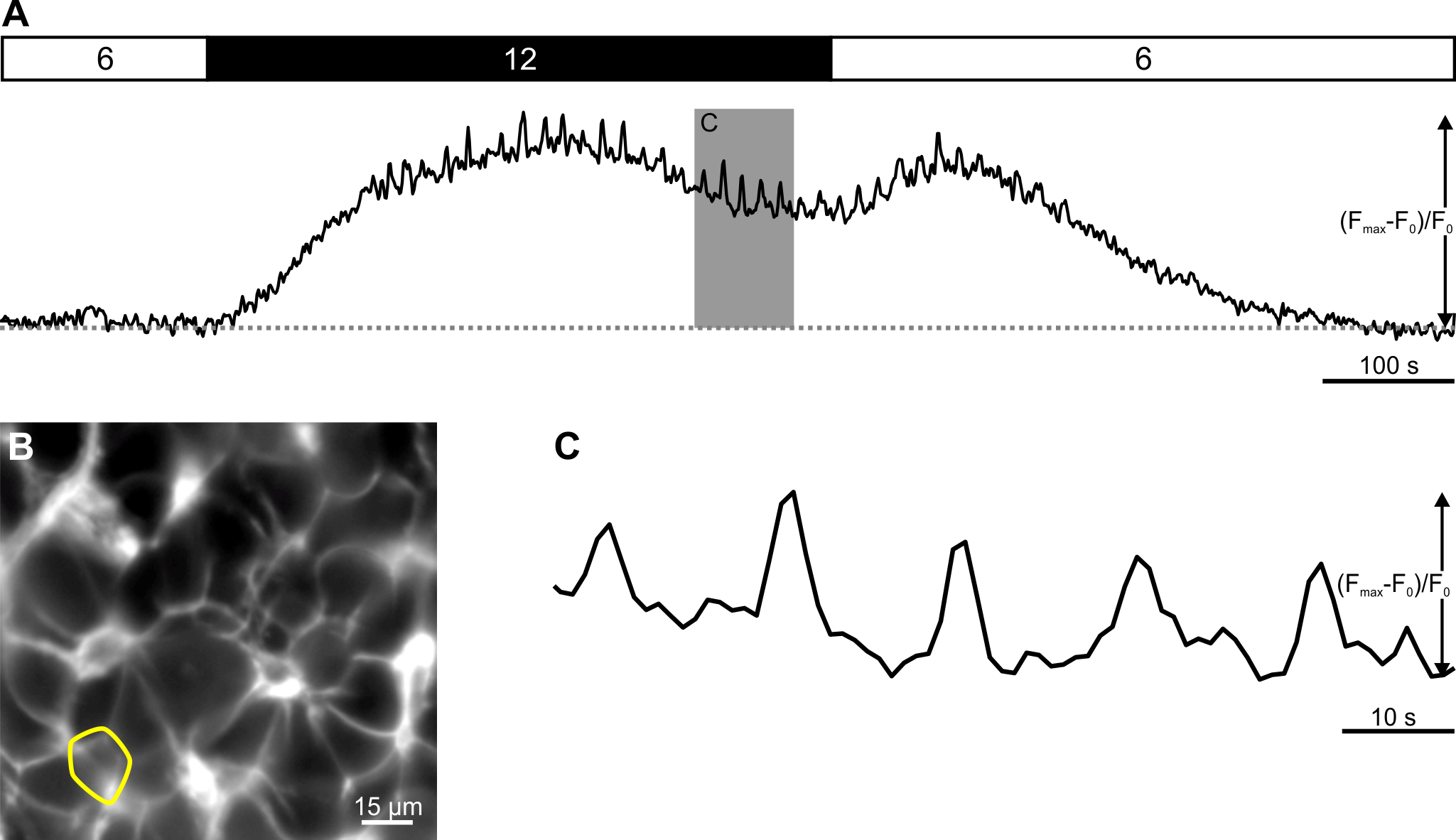

Supplement: Figure S1 — Membrane potential oscillations upon stimulation with 12 mM glucose. A Fluorescence trace of the voltage-sensitive dye VF measured with confocal imaging. Cell location within islet of Langerhans is marked in B. Numbers denote glucose concentration in mM. B Cell membranes in a representative islet of Langerhans are labeled with the voltage-sensitive dye VF. C Membrane potential oscillations indicated in panel A with the gray rectangular area are shown in detail. Y axis represents the normalized fraction of the difference between maximum and plateau baseline fluorescence. Sampling rate 1Hz at 512 x 256 pixels (from which 246 x 256 frame is shown). (TIF) [file pone.0082374.s001.tif]

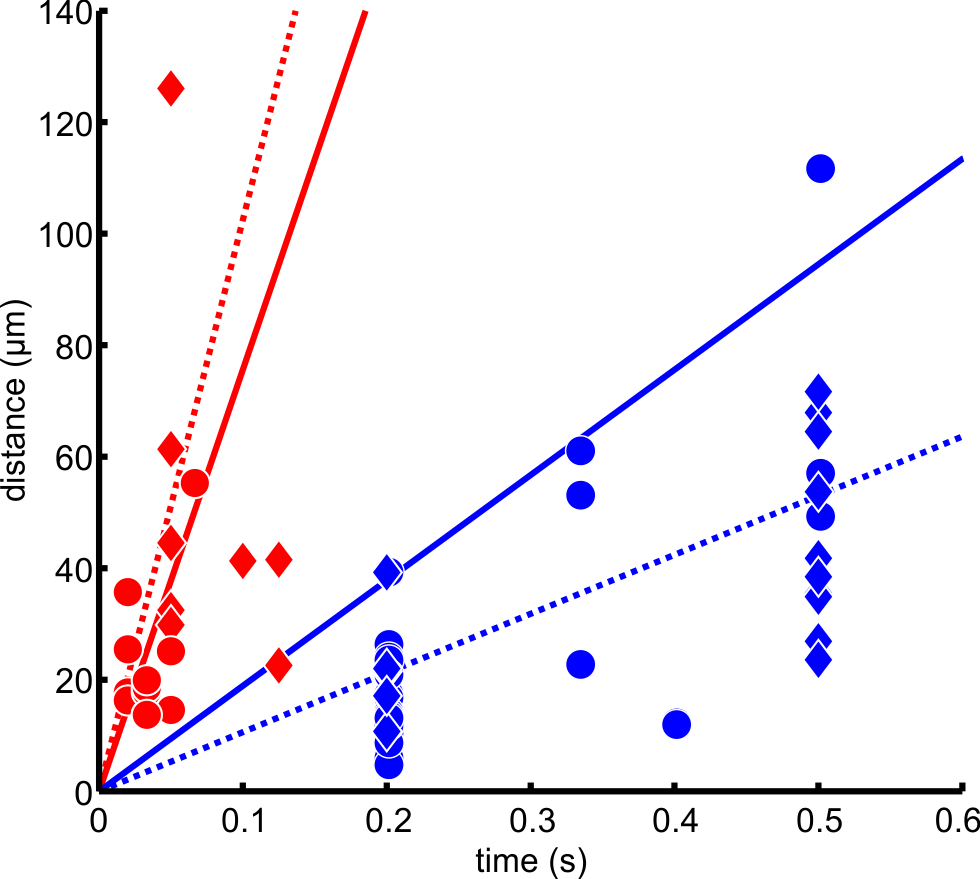

Supplement: Figure S2 — Velocities of [Ca]i and membrane potential waves during stimulation with 12 mM glucose and 12 mM glucose plus 10 mM TEA. Distance vs. time is plotted for the calculated trajectories during stimulation with 12 mM glucose (blue) and 12 mM glucose plus 10 mM TEA (red), assessed using the VF (circles) and the OGB-1 (diamonds) dye. The respective linear regression lines are drawn for the VF (solid line) and OGB-1 (dashed line) data during stimulation with glucose only (blue) and with glucose plus TEA (red). Note that the slopes of the regression lines directly represent the wave velocities: 188 and 106 µm/s for the VF and OGB trajectories during glucose only stimulations (27 data from 7 islets and 16 data from 6 islets, respectively) and 769 and 1031 µm/s for the VF and OGB trajectories during glucose plus TEA stimulation (12 data from 5 islets and 8 data from 3 islets, respectively). (TIF) [file pone.0082374.s002.tif]

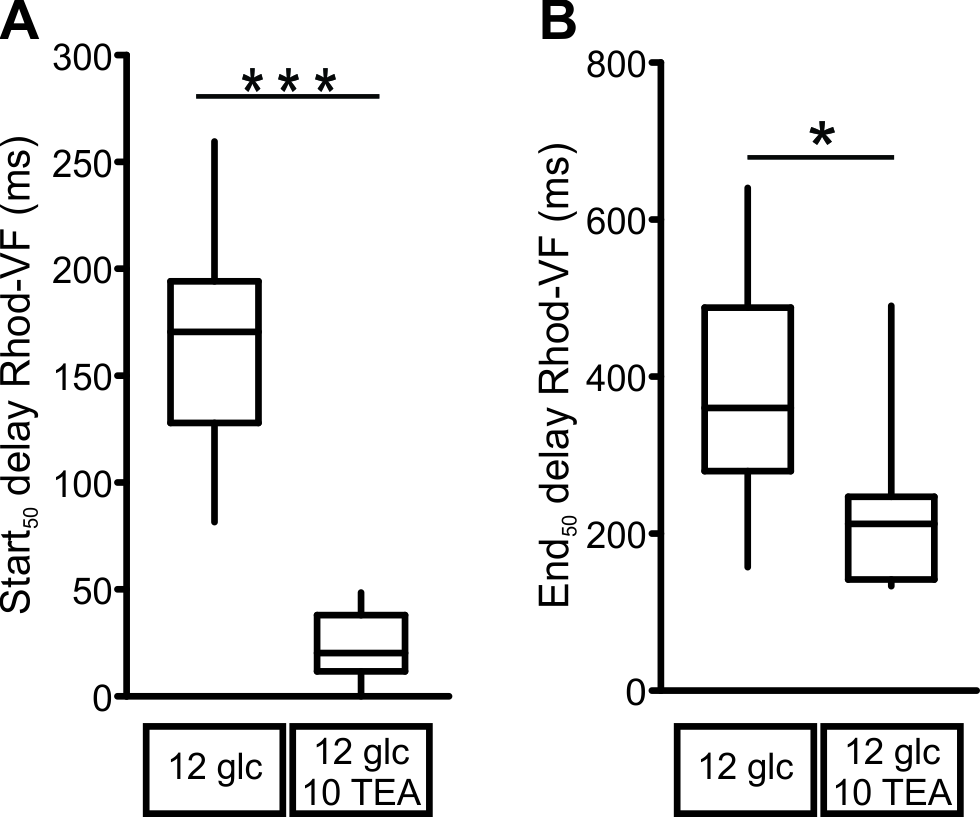

Supplement: Figure S3 — Delays between the Rhod-2 and VF signals of starts50 and ends50 during stimulation with glucose and glucose plus TEA. A During stimulation with 12 mM glucose the delay between starts50 of the Rhod-2 and VF signals (1st quartile=133 ms, median=170 ms, 3rd quartile=189 ms, n=21 cells) is statistically significantly longer than during stimulation with 12 mM glucose plus 10 mM TEA (1st quartile=13 ms, median=20 ms, 3rd quartile=36 ms, n=11 cells). Asterisks indicate p<0.001 (Mann Whitney test). B During stimulation with 12 mM glucose the delay between ends50 of the Rhod-2 and VF signals (1st quartile=284 ms, median=360 ms, 3rd quartile=473 ms, n=21 cells) is statistically significantly longer than during stimulation with 12 mM glucose plus 10 mM TEA (1st quartile=150 ms, median=212 ms, 3rd quartile=239 ms, n=11). Asterisk indicates p<0.05 (Mann Whitney test). (TIF) [file pone.0082374.s003.tif]

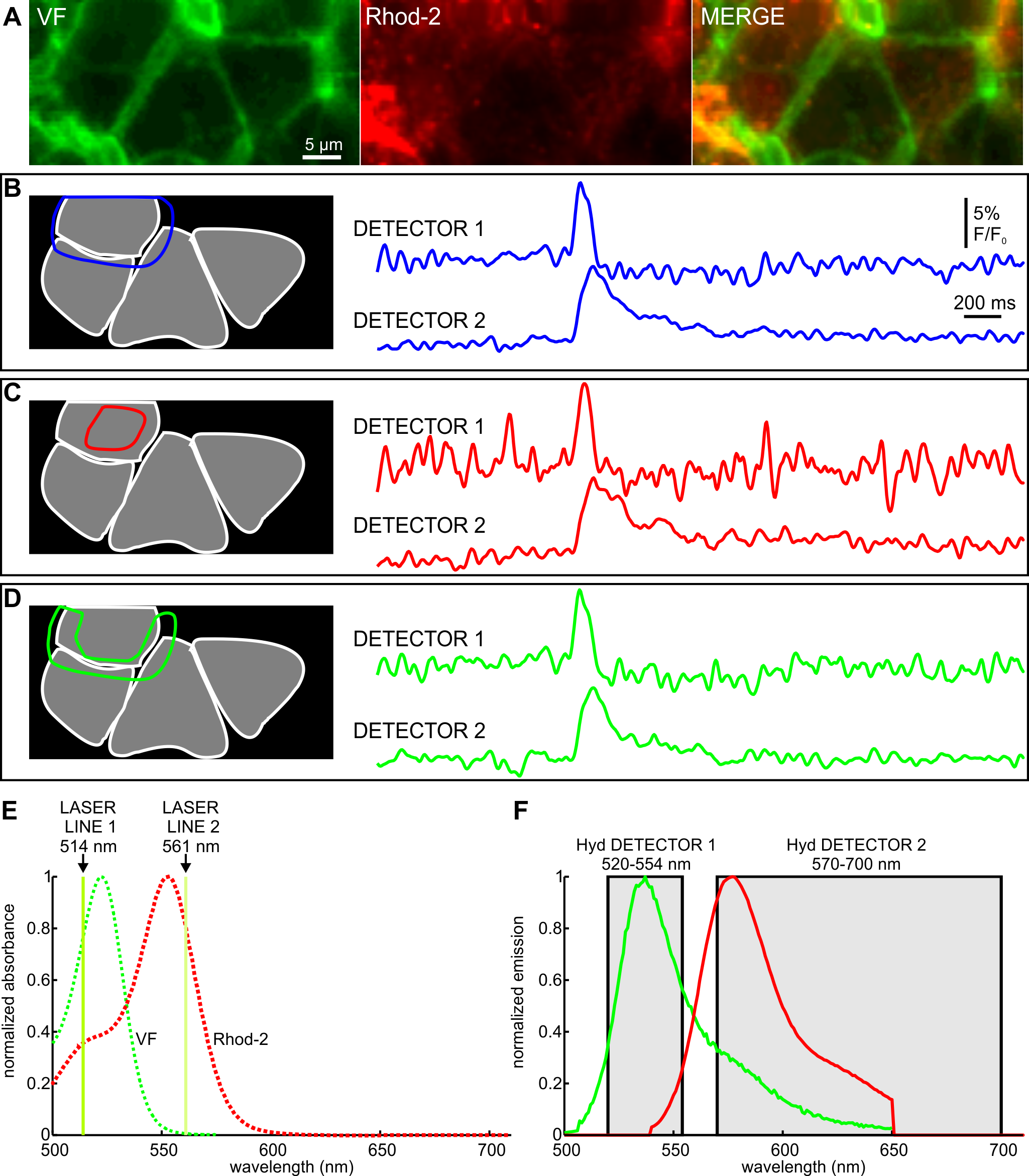

Supplement: Figure S4 — Experimental setup for simultaneous recording of membrane potential (using VF dye) and [Ca2+]i (using Rhod-2 dye). A VF (left panel) stained mostly cellular membranes, whereas Rhod-2 stained the cytoplasms (middle panel). For the active cells, no significant co-localization was observed (right panel). B-D Outlines of cells in A (left). Time traces for the VF and Rhod-2 are shown; the respective regions of interest are indicated on the left. Y axis represents the normalized fraction of the difference between maximum and plateau baseline fluorescence. E Absorption spectra for the VF and Rhod-2 dyes, indicated are the two laser lines used to excite the dyes. F Emission spectra for the VF and Rhod-2 dyes, indicated are the wavelength intervals in which emitted light was detected. Resolution was 128x64 pixels at 170 Hz. (TIF) [file pone.0082374.s004.tif]

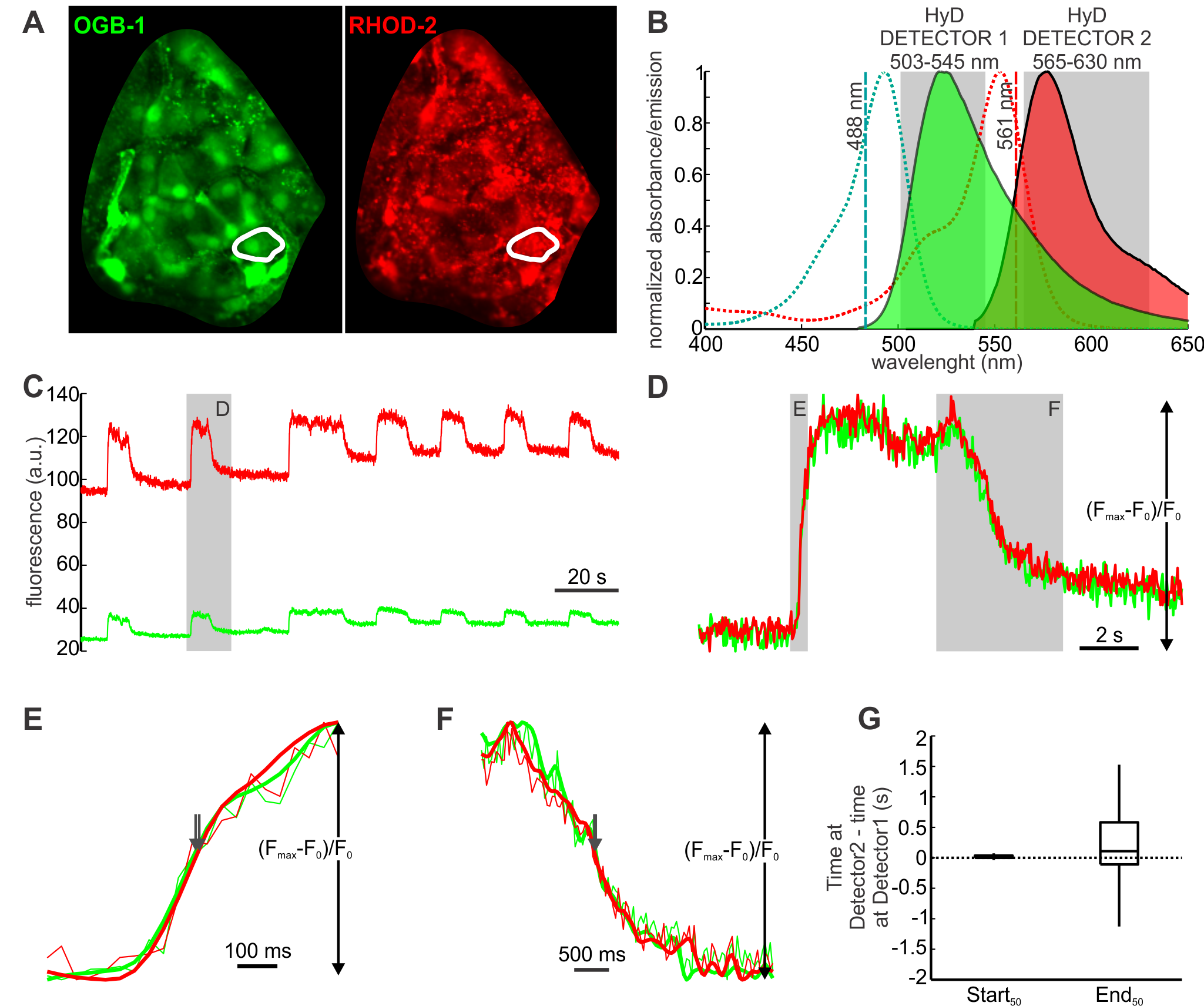

Supplement: Figure S5 — Comparison of [Ca2+]i dynamics measured with two different calcium indicators, OGB-1 and Rhod-2. A Slices were loaded with a loading mixture containing both OGB-1 AM and Rhod-2 AM. Indicated is a region of interest that is analyzed in C-F. B Excitation (broken line) and emission (solid line) spectra of OGB-1 (green) and Rhod-2 (red). Vertical broken lines indicate the 488 nm laser light exciting both dyes and the 561 nm laser light exciting Rhod-2 only. Detector 1 was set to measure OGB-1 signal only (0.4 % of the Rhod-2 emission is detected) whereas the detector 2 captured mostly Rhod-2 and partly OGB-1 emission (20 % of the total OGB-1 emission is detected with this detector). C 12 mM glucose elicited [Ca2+]i oscillations superimposed on the plateau phase of the response. Both detectors measured equal shapes of the seven [Ca2+]i oscillations. Note that the red signal is several times larger than the green signal at equal detector gains. In this and all other panels, signals from detector 1 and 2 are depicted in green and red, respectively. D A detailed representation of a single oscillation from C. [Ca2+]i dynamics of the green and red signals are identical. E The oscillation onset from panel D is shown in detail. For both the green and the red signals the starts50 of [Ca2+]i oscillations were determined at their half-maximal amplitude (indicated by arrows). Original (thin lines) and filtered data (thick lines) are shown. F End of the oscillation from panel D is shown in detail. Ends50 of [Ca2+]i oscillations were determined for the red and green signals at their half-maximal amplitude (indicated by arrows). Original (thin lines) and filtered data (thick lines) are shown. G Differences in starts50 and ends50 of [Ca2+]i oscillations of the red and green signals for the 7 oscillations in 9 cells. The difference between the two signals was not statistically significantly different from zero for starts50 and ends50 (p>0.05). (TIF) [file pone.0082374.s005.tif]

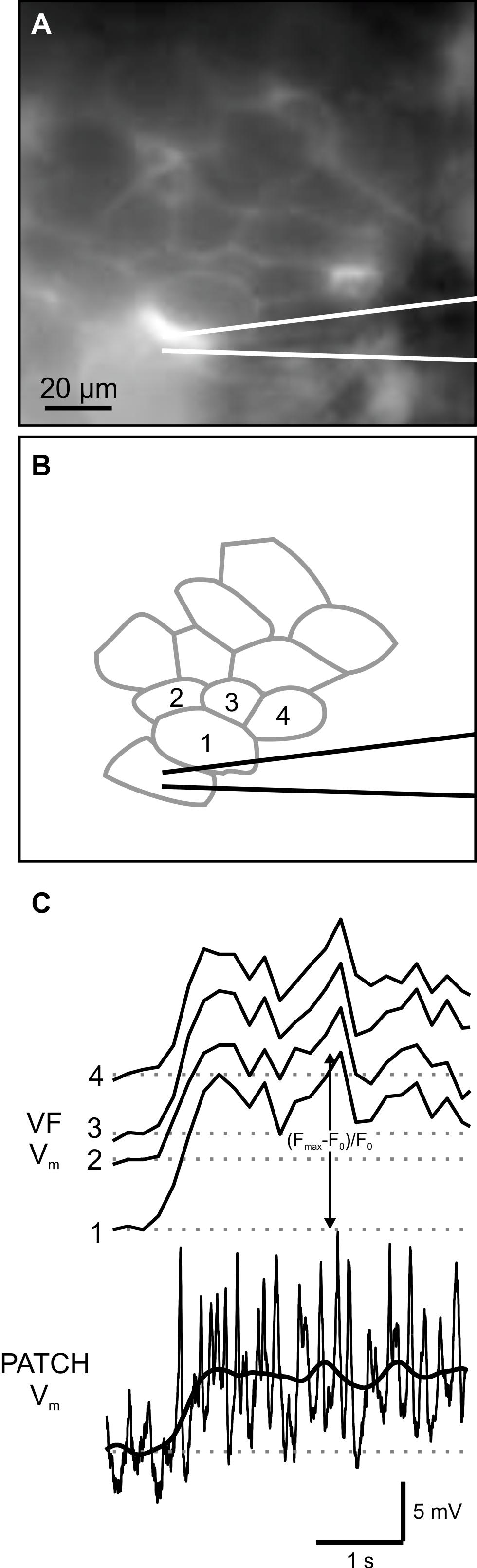

Supplement: Figure S6 — Simultaneous recording of membrane potential change with the VF dye and with the patch-clamp technique. A The VF dye loaded many cells in this islet of Langerhans. White lines indicate location of the patch-clamp pipette. B A scheme indicating active cells. The patch pipette is in contact with the cell from which the membrane potential was recorded with the whole-cell patch-clamp technique. Its neighboring cells from which the membrane potential was measured as VF fluorescence are marked with numbers. C In the patched-clamped cell the membrane potential depolarization was followed by superimposed spikes (lower trace marked with PATCH Vm). In addition to the raw signal, a smoothed signal of the membrane potential change is shown (thicker solid line). In the neighboring cells the VF fluorescence transiently increased to a new plateau value (traces marked with VF Vm). Note the equal timing and shape of the fluorescence signal compared to the smoothed signal recorded with the patch-clamp. The high frequency spikes are missing from the fluorescence signal due to sampling rate of the VF signal (5 Hz). Sampling rate for the patch-clamp was 1000 Hz. Traces represent the normalized fraction of the difference between maximum and plateau baseline fluorescence. The VF fluorescence was captured using the CCD camera at 256x256 pixels. (TIF) [file pone.0082374.s006.tif]
