# Supplementary material for: The Relationship between Membrane Potential and Calcium Dynamics in Glucose-Stimulated Beta Cell Syncytium in Acute Mouse Pancreas Tissue Slices
Source: PLoS One. 2013 Dec 6;8(12):e82374. doi: 10.1371/journal.pone.0082374 (PMC3855743; doi:10.1371/journal.pone.0082374)
Supplement: Text S1 — Substantiation of the Rhod-2 as indicator of cytosolic [Ca2+]. (DOCX) [file pone.0082374.s007.docx]

**Substantiation of the Rhod-2 as indicator of cytosolic [Ca^2+^]**

Rhod-2 was shown to be internalized into mitochondria due to its positive charge [[1](#_ENREF_1),[2](#_ENREF_2)]. In pancreatic acinar cells the intramitochondrial [Ca^2+^] change was shown to lag behind the cytosolic [Ca^2+^] [[1](#_ENREF_1)]. Therefore, in our case the observed time lags between the Rhod-2 and VF signals (Figures 6-7) could be an artifact of the inappropriate reporter dye.

There are several considerations worth mentioning that in our view strongly limit the above possibility.

1. Although Rhod-2 is known to target mitochondria due to its positive charge, Rhod-2 mitochondrial localization is not perfect – e.g. in rabbit myocyte it is primarily cytosolic [[2](#_ENREF_2)]. The localization of Rhod-2 to the mitochondria has not been studied in detail in beta cells.
2. The relative contribution of the signal from mitochondria in the thin optical sections used in our confocal experiments is small compared to the cell cross-section (refer to plate 6 in reference [[3](#_ENREF_3)]), therefore minimizing the contribution to the overall fluorescence.
3. During stimulation with glucose, the cytosolic [Ca^2+^] is expected to lie in the range of 50-200 nM with approximately 50 nM amplitudes of superimposed [Ca^2+^] oscillations [[4](#_ENREF_4),[5](#_ENREF_5)], whereas the mitochondrial Ca^2+^ concentration is probably > 4 µM [[6](#_ENREF_6)]. Such high [Ca^2+^] concentration lies at the upper limit of the Rhod-2 dynamic range (K_d_ = 570 nM), therefore this is an additional reason to believe that the contribution of mitochondrial [Ca^2+^] changes to the total fluorescence signal should be attenuated.
4. In beta cells, the mitochondrial [Ca^2+^] was reported to lag behind the cytosolic [Ca^2+^] oscillations by a few tens of seconds [[7](#_ENREF_7)], the lag being far beyond the time scale used in our analyses (< 500 ms).

Taken together, we believe that the possibility of mitochondrial [Ca^2+^] critically crosstalking with the cytosolic [Ca^2+^] is highly unlikely.

Nevertheless, we addressed this possibility also experimentally and included the results in the Figure S5. We double stained the tissue slices with OGB-1 and Rhod-2 (Figure S5). The OGB-1 dye is neutral in its charge and thus limited to cytosol and nucleus. The experimental setup was organized in such a way that detector 1 (green color in the Figure S5) reported the response of the OGB-1 dye only. This was confirmed by switching off the 488 nm laser (used to excite OGB-1), which completely abolished the signal in detector 1 (data not shown). Detector 2, on the other hand, picked up a composition of Rhod-2 and OGB-1 signals. Due to the known emission spectrum of the OGB-1 dye, the contribution of the OGB-1 signal to the detector 2 is a specific fraction of the detector 1 value. Since the red signal from detector 2 was several times larger than the green signal from detector 1 at equal detector gains, the majority of the signal originated from Rhod-2 emission.

Stimulation with 12 mM glucose revealed no difference in the temporal profiles between the green and the red signals at either the start or the end of the [Ca^2+^]_i_ oscillations (Figure S5D-F). Start_50_ and end_50_ time points of seven [Ca^2+^]_i_ oscillations were determined at half maximal amplitudes of each [Ca^2+^]_i_ oscillation (n=9 cells) separately for the green and the red signal (Figure S5G). Differences in time points at detector 2 and 1 were not statistically significantly different from zero for either start_50_ or end_50_ of the [Ca^2+^]_i_ oscillations. This corroborates our view that the findings presented in Figures 6 and 7 are not an artifact of our choice of Rhod-2 as the reporter for [Ca^2+^]_i_ changes.

**References:**

1. Johnson PR, Tepikin AV, Erdemli G (2002) Role of mitochondria in Ca2+ homeostasis of mouse pancreatic acinar cells. Cell Calcium 32: 59-69.

2. del Nido PJ, Glynn P, Buenaventura P, Salama G, Koretsky AP (1998) Fluorescence measurement of calcium transients in perfused rabbit heart using rhod 2. American Journal of Physiology-Heart and Circulatory Physiology 274: H728-H741.

3. Munger BL (1958) A light and electron microscopic study of cellular differentiation in the pancreatic islets of the mouse. American Journal of Anatomy 103: 275-311.

4. Bergsten P, Grapengiesser E, Gylfe E, Tengholm A, Hellman B (1994) Synchronous oscillations of cytoplasmic Ca2+ and insulin release in glucose-stimulated pancreatic islets. J Biol Chem 269: 8749-8753.

5. Gilon P, Shepherd RM, Henquin JC (1993) Oscillations of secretion driven by oscillations of cytoplasmic Ca2+ as evidences in single pancreatic islets. Journal of Biological Chemistry 268: 22265-22268.

6. Rutter GA, Theler JM, Murgia M, Wollheim CB, Pozzan T, et al. (1993) Stimulated Ca2+ influx raises mitochondrial free Ca2+ to supramicromolar levels in a pancreatic beta-cell line. Possible role in glucose and agonist-induced insulin secretion. J Biol Chem 268: 22385-22390.

7. Tarasov AI, Semplici F, Li DL, Rizzuto R, Ravier MA, et al. (2013) Frequency-dependent mitochondrial Ca2+ accumulation regulates ATP synthesis in pancreatic beta cells. Pflugers Archiv-European Journal of Physiology 465: 543-554.
